# Building consensus: construction of a juvenile and adult scRNA-seq meta-atlas for dataset comparisons and harmonizing transcriptomic definitions of enteric neuron subtypes

**DOI:** 10.1186/s12864-025-12283-5

**Published:** 2026-01-22

**Authors:** Joseph T. Benthal, Aaron A. May-Zhang, E. Michelle Southard-Smith

**Affiliations:** 1 Division of Genetic Medicine, Vanderbilt University School of Medicine, 2215 Garland Ave, 525 Light Hall, Nashville, TN 37232-0275 USA; 2 Vanderbilt University PhD Program in Human Genetics, Nashville, TN 37232 USA; 3 Illumina, San Diego, CA 92122 USA

**Keywords:** Enteric nervous system (ENS), Single cell RNA, Sequencing (scRNA-seq), Single nucleus RNA, Sequencing (snRNA-seq), Gene Expression Omnibus (GEO), Uniform manifold approximation projection (UMAP), Meta Analysis

## Abstract

**Background:**

The enteric nervous system (ENS) is a complex network of interconnected ganglia within the gastrointestinal (GI) tract. Among its diverse functions, the ENS detects bowel luminal contents and coordinates the passing of stool. ENS defects predispose to GI motility disorders. Previously, distinct enteric neuron types were cataloged by dye-filling techniques, immunohistochemistry, retrograde labeling, and electrophysiology. Recent technical advances in single cell RNA-sequencing (scRNA-seq) have enabled transcriptional profiling of hundreds to millions of individual cells from the intestine. These data allow cell types to be resolved and compared using their transcriptional profiles (“clusters”) rather than relying on antibody labeling. As a result, greater diversity of enteric neuron types has been appreciated. Because each scRNA-seq study has relied on different methods for cell isolation and library generation, numbers of neuron clusters and cell types detected differ between analyses. Cell counts in each dataset are particularly important for characterization of rare cell types since small numbers of profiled cells may not sample rare cell types. Importantly, each dataset, depending on the isolation methods, may contain different proportions of cells that are not detected in other datasets. Aggregation of datasets can effectively increase the total number of cells being analyzed and can be helpful for confirming the presence of low-abundance neuron types that might be absent or observed infrequently in any single dataset.

**Results:**

Here we briefly systematically review each *Mus musculus* single cell or single nucleus RNA-sequencing (snRNA-seq) ENS dataset. We then reprocess and computationally integrate these select independent scRNA-seq enteric neuron datasets from the myenteric plexus with the aim to identify new cell types, shared marker genes across juvenile to adult ages, dataset differences, and achieve some consensus on transcriptomic definitions of enteric neuronal subtypes.

**Conclusions:**

Data aggregation generates a consensus view of enteric neuron types and improves resolution of rare neuron classes. This meta-atlas offers a deeper understanding of enteric neuron diversity and may prove useful to investigators aiming to define alterations among enteric neurons in disease states. Future studies face the challenge of connecting these deep transcriptional profiles for enteric neurons with historical classification systems.

**Supplementary Information:**

The online version contains supplementary material available at 10.1186/s12864-025-12283-5.

## Background

The enteric nervous system (ENS) is a network of ganglia intrinsic to the gut wall that encircles the intestine and mediates peristalsis for passing of stool. The ENS, like the central nervous system, is made up of many distinct neuronal subtypes whose functions coincide with position along the intestine and specific roles in the circuitry of this system [1]. Historically, the cell types of the ENS have been defined by several techniques including dye-filling, immunohistochemistry, retrograde labeling, electrophysiology, and a variety of other methods [2]. From these studies, the ENS field has gained a broader understanding of how discrete cell classes function, allowing researchers to model functional deficits due to disease. Several studies have shown that the balance or complete absence of discrete subtypes of enteric neurons can occur in gastrointestinal (GI) motility disorders, including Hirschsprung disease [3–5]. Given the imbalance of enteric neuron subtypes in some GI motility disorders, it is important to understand particularly the diversity of neuronal cell types in the ENS so that regenerative medicine efforts can target the production of distinct neuron types.

Advances in single cell transcriptomics have enabled profiling of individual cells from an array of tissues including the ENS where efforts to define enteric neuron diversity previously relied on immunohistochemistry, dye-filling, or pharmacological studies of single neurons. To date, at least 15 distinct mouse ENS single cell or nucleus datasets have been produced by 14 different studies, with each publication using different isolation methods, strains/ages of mice, and methods of cell or nuclei isolation [5–19]. Several reviews have been published comparing the metrics of these datasets [2, 20, 21]. However, to our knowledge, no effort has been made to aggregate these data to gain greater insights into cellular diversity of the ENS. Each individual study profiling ENS progenitors and mature enteric neurons applied distinct technological and bioinformatic approaches to produce and analyze scRNA-seq or snRNA-seq data. Variables included differing mouse lines, ages, tissue dissociation methods, techniques for encapsulation/library production, and depth of sequencing [5–19]. These differences have led to variation in the number of cells sampled, cell types detected, and genes whose expression labels distinct cell clusters [2, 20, 21]. A main point of discussion in the ENS field centers on reaching consensus regarding how many ENS cell types exist and what methods should be prioritized for classifying and naming them for consistency across the literature. Combining enteric neuron scRNA-seq datasets in a strategic manner is one means to gain greater understanding of diversity of enteric neurons. In this work, we first systematically review each published scRNA-seq and snRNA-seq dataset derived from the ENS, highlighting the main contributions to the field that each work produced. We then make the case for combining the more mature enteric neuron datasets and illustrate the integration process, outlining both advantages and caveats for this method to further refine transcriptional states of enteric neurons. Our goal is to demonstrate how meta-analysis (the process of pooling independent datasets together into one larger aggregate) can yield consistent cell states across datasets, a higher sample size of enteric neurons and therefore greater resolution to detect rare cell states. We offer this resource of similarly processed datasets for future mining by the ENS community.

## Results

### Overview of ENS single cell RNA-sequencing studies and the numerous neuron types revealed

Many single cell studies on the enteric nervous system have been published with various goals including defining neuronal subtypes, development of those subtypes, and/or how these are altered in knockout or disease models. Here, we first review the dataset features and major findings of recent single cell/nuclei profiling studies as a prelude to our main goal of data aggregation for refined understanding of enteric neurons.

Lasrado and colleagues produced the first ENS scRNA-seq dataset [8]. The study aimed to define spatial coordination of murine ENS development by utilizing a *Sox10-creER*^*T2*^ transgenic activating a ROSA confetti reporter at 12.5 days post coitus (dpc). The analysis identified spatially distinct bipotent and fate-restricted progenitors/precursors originating from neuroblasts with limited proliferative capacity and glial progenitors with higher proliferative capacity. Using fluorescence activated cell sorting (FACS) the team isolated confetti-tagged ENS cells at 12.5–13.0 dpc for scRNA-seq libraries. While these experiments produced a small dataset containing 120 cells, the analysis identified three cell clusters distinguished by expression of typical glial (*Erbb3*, *Sox10*, *Fabp7*), progenitor (*Plp1)* and neuronal (*Tubb3*, *Elavl4*, *Ret*) marker genes. The expression profiles were consistent and further refined earlier reports of neuronal and glial lineage divergence during colonization of the fetal mouse intestine and offered an initial view of the genes being transcribed at this critical stage in ENS development [22].

A second ENS scRNA-seq dataset was part of a larger mouse nervous system atlas from [13], in which mouse ENS cells were collected from postnatal (P) stages [13]. This study relied on lineage tagging of ENS populations from *Wnt1-Cre;ROSA26tdTomato* reporter mice for fluorescent postnatal labeling of neural crest derivatives at P19, P20, and P21 (Table 1). Generation of tissue dissociates from younger mice is easier than in older animals, and the ages collected were designed to profile changes in the ENS that occur around the time of weaning (P20-P21) when animals transition to solid food. In this effort, analysis of the resulting scRNA-seq libraries found most transcriptional profiles originated from enteric glia. This outcome most likely resulted from loss of membrane integrity among neurons during tissue dissociation, which is known to shear neuronal processes, and could allow uptake of viability dyes leading to cells being rejected during flow sort isolation [23, 24]. Despite this challenge, the team successfully profiled 727 enteric neurons that produced nine cell clusters in t-SNE representations using typical clustering algorithms. Overall, the analysis found gene expression patterns typical of glutamatergic, cholinergic, and nitrergic enteric neuron types.

**Table 1:** Characteristics of ENS RNA-sequencing datasets

First Author	Year	Stage (dpc)	Mouse Model	Genetic Background	Genotype	Cell or Neuron Counts
Lasrado	2017[8]	13; Tam 12.0	*Sox10*-*creERT*^*2*^(SER93);*R26R-tdTomato*	129S1/Sv x 129X1/SvJ x 129S6/SvEvTac x C57BL/6NCr	WT	120
Zeisel	2018[13]	P19, P21, P23	*Wnt1-cre*;*R26RTomato*	C57Bl6J	WT	727 ENS neurons
Lau	2019[9]	13.5	*Wnt1*-*cre*;*YPF*, *Tg*(*GBS-GFP*)	C57 × 129/S6, mixed outbred	WT	*Wnt1*-*cre*;*YPF*: 7671, *Tg*(*GBS-GFP*): 3858
Drokhlyansky	2020[6]	P77-728	*Sox10-cre*;*INTACT*, *Wnt1-cre2*;*INTACT*, *Uchl1*-*H2BmCherry:GFP-gpi*	C57BL/6 J, (C57BL/6 × CBA)F1, C57BL/6 × C3H, 129, (C57BL/6 × SJL)F2	WT	RAISIN 2657; MIRACL 2411
Lai	2021[7]	13.5	*Wnt1*-*Cre*;*Rosa26*^*YFP*^, *Wnt1-Cre*;*Kif7*^*f/f*^;*Rosa26*^*YFP*^	C57 × 129/S6, mixed outbred	WT, *Kif7* conditional KO	Control 7671; Mutant 15,522
May-Zhang*	2021[10]	P42-47	*Phox2b-*H2BCFP	C3HeB/FeJ x C57BL/6 J F1	WT	18,547
Morarach	2021[11]	15.5	*Wnt1-cre;R26R-tdTomato*	Unspecified, 129S6/SvEvTac, C57BL/6NCrl mixed background	WT	3260
Morarach	2021[11]	18.5	*Wnt1-cre*;*R26R-tdTomato*	Unspecified, 129S6/SvEvTac, C57BL/6NCrl mixed background	WT	2733
Morarach	2021[11]	P21	*Baf53b-cre*;*R26R-tdTomato*	C57BL/6, 129S6/SvEvTac, C57BL/6NCrl mixed background	WT	4892
Wright	2021[12]	17.5	*Chat*-*EGFP-L10A* +; *Nos1-creERT2*^*/*+^;*R26R-tdTomato*	C57BL/6 J	WT	707 neurons
Wright*	2021[12]	P47–52	*Wnt1-cre*;*R26R-H2BmCherry*	(C57BL/6 J x CBA/J)F1 x (129S4/SvJaeSor x C57BL/6 J)F1	WT	635 neurons
Bhave	2022[5]	P14	*Wnt1-tdT;Ednrb*^*−/−*^	129S7/SvEvBrd, C57BL/6 J, CBA/J, 129S6/SvEvTac, C57BL/6NCrl mixed background	*Ednrb* KO	2060
Guyer	2023[14]	P14	B6;CBA-Tg(Plp1-EGFP)10Wmac/J	C57BL/6 × CBA	WT	17,690 enteric glial cells
Vincent	2023[15]	12.5, 14.5	*Ret*^*CFP/*+^, *Ret*^*CFP/CFP*^	C57BL/6	*Ret* null, *Ret* heterozygous mutant	1003
Kulkarni	2023[16]	P21; ~ P180	C57BL/6	C57BL/6	WT	Neural-crest-derived: ~ 1300, putative mesoderm-derived: ~ 500; Neural-crest-derived: 1737, putative mesoderm-derived: 2223
Schneider	2024[17]	P5	*TyrBap1* *R26R-TdTomato* (KO)* and Bap1-wt/wt, Tyr-Cre R26-TdTomato*	C57BL/6	WT, *Tyr-* *Bap1* KO	WT: 1392; *Tyr-* *Bap1*: 2382
Zhou	2024[18]	10.5, 12.5, 14.5, 17.5, P21	*Sox10-CreER T2;Rosa26R-EGFP* (No FACS)	C57BL/6, CBA/J, 129S4/SvJaeSor mixed backgrounds	WT	4741
Li	2025[19]	P7	*Wnt1-Cre;R26RtdTom*	Unspecified, 129S6/SvEvTac, C57BL/6NCrl mixed background	WT	5740
Li	2025[19]	P24	*Baf53b-Cre; R26RtdTom*	Unspecified, 129S6/SvEvTac, C57BL/6NCrl mixed background	WT	8341

^*^These studies utilized single nucleus RNA-sequencing

Lau and colleagues subsequently produced an extensive fetal mouse ENS scRNA-seq dataset at 13.5 dpc while analyzing Hedgehog signaling [7, 9]. This team also relied on the *Wnt1-cre*;Rosa26*YFP* to isolate 7671 neural crest cells from fetal mice. They compared their mouse transcriptional profiles to scRNA-seq from human pluripotent stem cell (hPSC)-derived neural crest derivatives. From principal component analysis, the group identified 8 mouse and 12 human cell clusters. Like the Lasrado study, Lau and colleagues also observed two distinct lineages for enteric neurons and glia. They then captured additional enteric lineages for scRNA-seq by utilizing a GLI1-3 GFP fluorescent reporter mouse line, *Tg*(*GBS-GFP*), that mirrors hedgehog signaling levels. From this effort, they produced transcriptional profiles from 2017 Hedgehog “on” and 1841 Hedgehog “low” signaling cells. Integration of these cells into their original 7671-cell scRNA-seq dataset found these cells are distributed across all 8 mouse cell clusters, which suggests GLI1-3 activity is dynamic across ENS differentiation.

Optimization of cell isolation methods and new informatic tools led to two independent studies in quick succession that compared scRNA-seq data from adult ENS of mice and humans authored by Drokhlyansky et al. and May-Zhang et al. [6, 10] Drokhlyansky and colleagues transcriptionally profiled adult mouse and human ENS from older cancer patients at single cell resolution [6]. This team relied on two complementary strategies that facilitated transcriptional profiling of enteric neurons via snRNA-Seq. The first strategy consisted of a new technique for isolation of nuclei with bound ribosomes on the outer nuclear membrane called Ribosomes and Intact single nucleus (RASIN) RNA-Seq that generated sequence data with higher spliced mRNA recovery than RNA-Seq of nuclei alone. The second strategy, Mining Rare Cells sequencing (MIRACL-seq), informatically selected droplets containing rare cells from overloaded single cell encapsulations generated from human bowel. For comparison of human enteric neurons with those of mice, the team isolated ENS cells from adult mice of both sexes ranging from 11 to 104 weeks of age. Transgenic lines that fluorescently tagged nuclei of neural crest lineages (*Sox10-cre:INTACT, Wnt1-cre:INTACT*) or enteric neurons (*Uchl1-H2BmCherry:GFP-gpi*) were used for flow sorting to enable transcriptional profiling of 2657 neurons and 3039 glia from colon. The authors identified colonic neuron clusters that putatively represent 21 distinct neuron types consisting of excitatory and inhibitory motor neurons, interneurons, sensory types, and secretomotor/vasodilator neurons as well as three enteric glial types. The analysis indicated regional differences in the distribution of enteric neuron types in the mouse colon that also varied by age and circadian phase. The Drokhlyansky team then applied MIRACL-seq to characterize mouse and human enteric neurons. This strategy captured transcriptional profiles of 1938 mouse neurons that parsed into 18 neuron types with some notable differences detected between colonic and ileal neurons. Similar MIRACL-seq profiling of muscularis propria from human colon cancer patients ranging in age from 35–90 years identified 1,445 neurons that segregated into 14 neuron classes. By comparing the mouse RNA-seq data to the human MIRACL-seq profiles the authors concluded that multiple types of enteric neurons are transcriptionally similar between species.

Comparison of transcriptional profiles between species and subsequent in situ localization of neuronal subtype markers in mice and humans performed by May-Zhang and colleagues reached slightly different conclusions [10]. This team aimed to compare enteric neuron gene expression between healthy young adult humans (18–35 years of age) and young adult mice (6–7 weeks of age). Their efforts initially focused on capture of myenteric neuronal nuclei from duodenum, ileum, and colon based on *Phox2b*-H2BCerulean transgene expression that is highly expressed in differentiated neurons [25]. The team successfully captured more than 25,000 nuclei with retention of 18,500 profiles after quality control criteria were applied. Clustering of these data generated 15 distinct clusters that parsed into 22 neuron types. These 22 neuron types were present across all three intestinal regions studied. Instead of attempting to flow sort neurons or neuronal nuclei from human intestine, the group relied on laser capture microdissection of histochemically stained myenteric ganglia collected from healthy young organ donors. The team used comparative analysis to remove genes expressed in surrounding muscle, producing a deep bulk dataset specific to human myenteric ganglia. By comparing genes expressed in their human bulk RNA-seq data to those present in mouse myenteric neurons, the authors were able to identify genes that marked distinct types of enteric neurons in both species. The regional and cell type specific expression of these “marker genes” were assessed in situ via hybridization chain reaction to localize distinct neuron types in each bowel region for both species [26, 27]. Importantly, the May-Zhang study implemented a method for blocking lipofuscin auto-fluorescence that is problematic in visualization of human neurons and can confuse interpretation of labeling [28]. The team’s analysis focused on localization of intrinsic primary afferent neurons (IPANs) in situ based on markers of somatostatin and calbindin-2 in parallel with Kelch Like Family Member 1 (*KLHL1*) present in murine IPANs. The team found that there is incomplete congruence of expression for genes that mark neuron types in mice compared to those expressed in human enteric neurons. Moreover, these authors determined that multiple genes exhibited regional differences in expression between the duodenum, ileum, and colon of mice, and most of these regional expression patterns were not detected in human tissues. These findings illustrate some cross species congruence, yet point to species distinctions, indicating the need for caution when extrapolating studies in mice for comparisons with human ENS studies.

Complementary ENS profiling from [11] produced transcriptional signatures from enteric populations in small intestines of mice as fetal progenitors differentiated towards neuronal fates [11]. Morarach and colleagues generated scRNA-seq profiles from neural crest-derived enteric neurons and glia based on *Wnt1-Cre:R26R-Tomato* expression with sequencing of 3260 cells at 15.5 dpc and 2733 cells at 18.5 dpc. To assess murine juvenile enteric neuronal diversity, the team utilized a *Baf53b-Cre* transgene that labels large numbers of enteric neurons to gain transcriptional profiles of an additional 4892 cells from the myenteric plexus of the small intestine at P21. From these data, the authors defined 12 main transcriptional enteric neuron cell (“ENC”) states that mostly express either *Etv1* or *Bnc2* in a dichotomous manner. The authors rigorously validated all detected neuron types via immunohistochemistry for marker genes in each cluster. Their validation studies showed that two neuron subsets exhibit morphological features consistent with known morphology of IPANs. In their fetal datasets, the authors reported the same *Etv1*, *Bnc2* transcriptional dichotomy in two “branch” trajectories of emerging enteric neurons over developmental time. This distinctive early branching in development of enteric neuron lineages contrasts with neurogenesis programs in the central nervous system and indicates that neuronal diversity in the ENS is elaborated post-mitotically. From their transcriptional profiling of developing ENS, Morarach and colleagues noted expression of *Pbx3*, a transcription factor expressed at a transition point bordering two neuron clusters in their scRNA-seq data. They further showed that loss of this gene produced altered ratios of Calbindin + neurons. The team’s work illustrates how careful attention to emerging transcriptional programs can identify key regulators that establish normal neuronal diversity in development.

Later, a study from Wright et al., 2021 [12] produced snRNA-seq data for mouse enteric neurons and glia from young adult mouse intestine based on labeling with *Wnt1-CreERT2*^*Cre/WT*^*:R26R-H2BmCherry* + crosses [12]. The team analyzed numerous mouse reporter lines to identify their selected combination that produced bright labeling of ENS nuclei and avoided erroneous flow sort gating due to adherence of neurites and cellular fragments attached to negative nuclei/cells that can occur with cytoplasmic tdTomato labeling. The analysis identified seven distinct neuronal clusters from 635 adult neurons sampled. Wright and colleagues further profiled fetal cholinergic (*ChAT-EGFP-L10AD* +) and nitrergic (*Nos1-CreERT2*^*Cre/WT*^*;R26R-TdTomatoD* +) neurons producing scRNA-seq data for 707 neurons that distributed into 8 distinct clusters at 17.5 dpc. The authors noted multiple differentially expressed genes between neuron types were known regulatory factors and utilized conditional gene deletion strategies to assess whether deletion of individual genes altered abundance of myenteric neuron types. The team’s analysis demonstrated that *Tbx3* is required in the fetal ENS for normal abundance of Nos + neurons. In contrast, deletion of other transcription factors (*Casz1*, *Tbx2*, and *Rbfox1*) from developing ENS did not alter abundance of cholinergic neurons. These studies suggest that production of some neuron subtypes may rely on more than one regulatory factor, a key finding for future directed differentiation of enteric neuron classes.

In 2022, Bhave and colleagues utilized scRNA-seq to assay *Ednrb*^*−/−*^ (knockout) P21 ENS cells from the small intestine to determine how this Hirschsprung mouse model might compare to the P21 scRNA-seq dataset of enteric neurons [11] [5]. Through this comparison paired with immunohistochemistry validation, the authors found that *Ednrb*^*−/−*^ mice were missing *Gad2*/*Neurod6* + enteric neurons in the small intestine. This was the first demonstration of a Hirschsprung mouse model lacking a specific ENS neuron subtype.

More recently, Guyer and colleagues in 2023 produced single cell multiome sequencing data from 17690 enteric glia from *Plp1::GFP* mice at P14 [14]. In addition, they also produced single cell RNA-seq data and single cell multiome sequencing for *Plp1::GFP* neurospheres derived from 12–14 week old mice. Using these data paired with immunofluorescence and RNAscope imaging, the authors confirm that enteric glial cells within ganglia are poised for undergoing neurogenesis in postnatal and adult stages.

In 2023, Vincent and colleagues produced a developmental ENS dataset comprised of *Ret*^*CFP/*+^ and *Ret*^*CFP/CFP*^ (loss of *Ret*) mutant cells [15]. They utilized this dataset to probe how the ENS develops in the absence of *Ret* expression, which they found affects the inhibitory neuron subtype differentiation, the timing required for proper neuronal and glial fate decisions, and ENS cell cycle dynamics.

Also in 2023, Kulkarni et al. published in *eLife* scRNA-seq data from longitudinal muscle-myenteric plexus (LM-MP) cells of mice at P10-30 and 6 months of age. This analysis relied on detecting ENS cells by gene expression markers instead of enrichment via flow sorting [16]. Based on immunohistochemistry data and cre-fate mapping supplemented by scRNA-seq, the authors demonstrated the presence of adult enteric neurons in the myenteric plexus that arise from a non-neural crest lineage. The authors concluded that these non-neural crest enteric neurons increase in proportion within the ENS as both mice and humans age. This particular scRNA-seq dataset was sequenced relatively shallowly and the authors concluded that these non-neural crest enteric neurons express genes suggestive of a mesodermal origin. This is the first study to document a population of enteric neurons that originates from a lineage independent of the vagal neural crest.

More recently, Schneider and colleagues in 2024 performed scRNA-seq of ENS at P5 to assess the effects of deleting the *Bap1*,a chromatin modifier, on ENS cell types [17]. Utilizing *Tyrosinase-Cre Bap1*^*fl/fl*^ mice, the authors found loss of *Bap1* in fetal stages caused a shift in enteric neuron subtypes, that was not evident when the gene was deleted in postnatal stages. This study reveals another gene that is required for proper subtype proportions, similar to *Sox10* as reported by Musser and colleagues in 2015 [3].

Also in 2024, Zhou and colleagues performed scRNA-seq on three gastrointestinal regions at five developmental stages in an effort to assay cells that represent the migrating wavefront of enteric neural crest-derived cells [18]. From the stomach, small intestine, and colon at 10.5, 12.5, 14.5, 17.5 dpc, and P21, they assayed a total of 4741 ENS cells. Based on differential gene expression and tracking over tissue section and time, the authors report a set of genes and a cluster of cells that are specifically upregulated in the migrating wavefront. The authors utilize spatial transcriptomics via Stereo-seq on the mouse gut segment containing the migrating wavefront at 12.5 dpc, to locate enrichment of the upregulated genes from their wavefront scRNA-seq clusters in the Stereo-seq wavefront.

Most recently, Li and colleagues in 2025 used scRNA-seq to assay the submucosal plexus at P7 and P24 to capture development and neuronal diversity of the submucosal ganglia (Meissner's plexus) [19]. This is the first time the submucosal plexus has been profiled, as most most other ENS scRNA-seq datasets only assay the myenteric plexus due to the relative ease of collecting the myenteric ganglia. The authors found three main submucosal enteric neuron types within the submucosa, fewer than have been identified in the myenteric plexus, including one unexpected type of IPAN.

Each study profiling ENS progenitors and mature enteric neurons applied distinct approaches both technological and bioinformatic to produce single cell or single nucleus RNA-seq data. Variables included differing mouse lines, ages, tissue dissociation methods, techniques for encapsulation/library production, and depth of sequencing (summarized in Table 1) [5–19]. These differences have led to variation in the number of cells sampled, cell types detected, and cluster-specific marker gene expression [2, 20, 21]. A main point of discussion in the ENS field centers on reaching consensus regarding how many ENS cell types exist and what methods should be prioritized for classifying and naming them for consistency across the literature. Combining enteric neuron scRNA-seq datasets in a strategic manner is one means to gain greater understanding of diversity of enteric neurons. We make the case below for this strategy and illustrate the process, outlining both advantages and caveats. Our goal is to demonstrate how meta-analysis (the process of pooling independent datasets together into one larger aggregate focused on the same question) can yield greater consistency of interpretation and offer a resource of similarly processed datasets for future mining by the ENS community.

### Why combine? The case for a meta-atlas of ENS scRNA-seq data

**Fig. 1:**
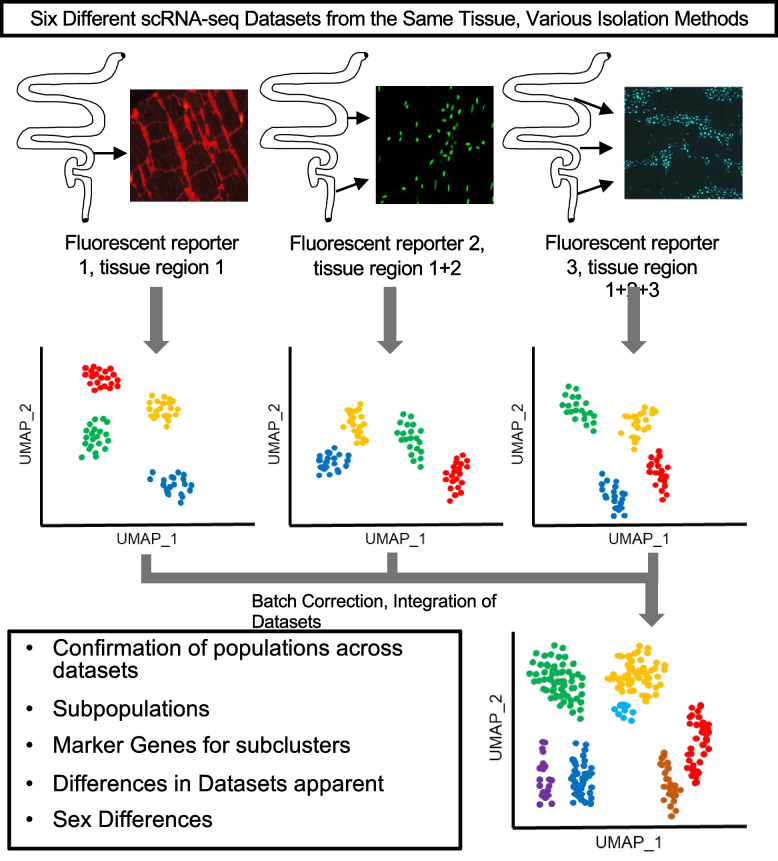
Overview of data integration process and analysis approach

In scRNA-seq experiments, sampling of each cell type within a tissue can be challenging, especially when those cells are rare. This is particularly the case for the ENS, where enteric neurons are embedded within the bowel wall, making up a low percentage of total cell numbers in the tissue. Low abundance is further complicated by difficulties in tissue dissociation and loss of neuronal processes due to shear forces during isolation. Even after successful cell isolation, differences in cell numbers profiled and the labeling strategy used to fluorescently tag enteric neurons for flow sort isolation contribute substantially to differences between resulting datasets. Recent success in combining multiple independent scRNA-seq datasets into a single aggregate, or “meta-analyses”, illustrate how combinatorial approaches have been informative for studies of COVID patients, liver homeostasis, and atherosclerotic tissues [29–31]. Each of these studies leveraged similarities across individual datasets and the increased cell numbers resulting from the integration to ask questions pertaining to the cell types of interest. They also successfully derived a consensus of cell type classifications and found novel cell subtypes that were not previously characterized from the individual datasets alone. Likewise, we take advantage of the similarities between ENS scRNA-seq datasets to successfully compile a “meta-atlas” of enteric neurons from the myenteric plexus that offers a deeper resource for discrimination between enteric neuron types. This approach (Fig. 1) reveals both commonalities between datasets as well as some differences between neuron types that were not as evident until data aggregation was performed.

### Advantages of reprocessing scRNA-seq data

Comparably processed datasets are optimal for production of a meta-atlas. Typically, initial scRNA-seq data in its large FASTQ file format is aligned via tools like CellRanger that produce barcode, matrix, and gene files [32]. These outputs are then imported into programs like Seurat for filtering, normalization, scaling, and dimensionality reduction to visualize differentially expressed genes [33–37]. It is possible to obtain aligned files associated with each publication although these often include alterations that result from data processing. Some authors provide the completely processed Seurat object; however, these files are too large for easy sharing. Importantly, use of derivative Seurat objects for comparisons can be complicated by how each dataset was processed. Differences in quality control metrics or clustering parameters can cause cells to be dropped from further analysis, lead to retention of poor-quality cells, or cause cell clustering differences. Reprocessing aligned data, such as that found in raw counts matrices and using a consistent set of parameters avoids these complications and leads to greater confidence in the outcomes produced by cross-study comparisons.

To illustrate how a meta-atlas can be produced, we primarily relied on semi-processed data files (such as CellRanger outputs) from alignment to the mouse genome (version mm10). We focused our ENS meta-analysis on juvenile and adult (P19 and older) ENS scRNA-seq datasets from the mouse myenteric plexus, as these cell states are most similar (Table 1). In contrast, the developmental datasets, despite expressing pan-neuronal marker genes, contain immature progenitor populations that are less likely to align with mature neuron clusters and can exhibit transient expression of developmental genes that are not retained in mature neuron types. We sourced the juvenile and adult data from either the Gene Expression Omnibus (GEO), the Single Cell Portal at the Broad Institute, or from mousebrain.org [6, 10–13, 38]. Some of the Drokhlyansky datasets had been pre-processed to eliminate heterotypic cells (sequenced droplets that contain two or more cells or nuclei of different types) using MIRACL-seq algorithms. We relied on these processed datasets for convenience, as most groups interested in utilizing these datasets may not have the computational tools for genome alignment. While not perfect, this is a straightforward approach for many groups that do not have advanced bioinformatics capabilities.

### Combining datasets: process, gains, and caveats

#### Reprocessing each sc/snRNA-seq dataset

**Fig. 2:**
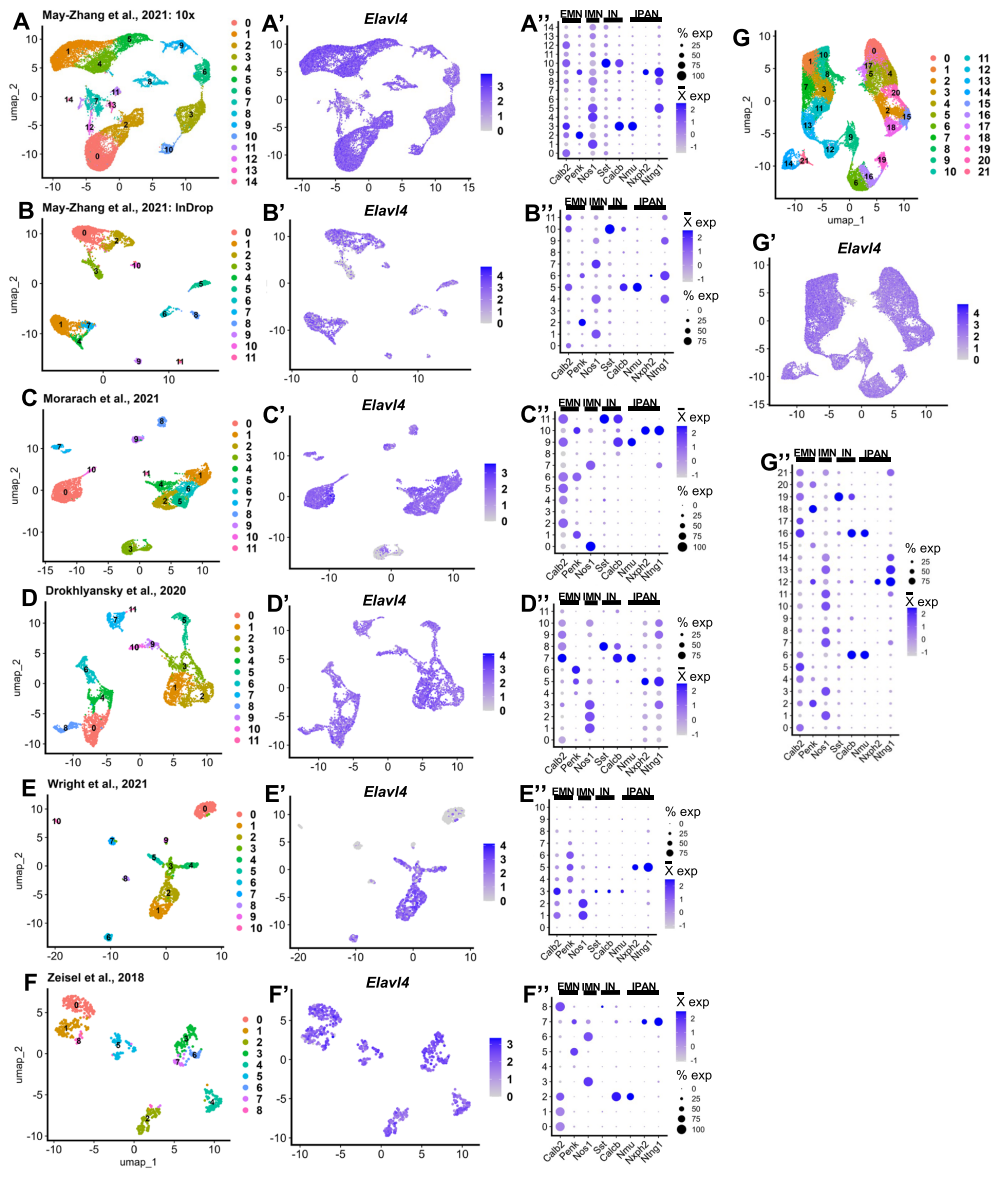
General consensus of enteric neuron types distributed across each single cell dataset. Panels **A**, **B**, **C**, **D**, **E**, and **F** show UMAP plots for each ENS dataset. Panels **A**’, **B**’, **C**’, **D**’, **E**’, **F**’, and **G**’ display expression of pan-neuronal marker *Elavl4* on UMAPS of each dataset. Panels **A**’’, **B**’’, **C**’’, **D**’’, **E**’’, **F**’’, and **G**’’ display dot plots of established marker genes detected in enteric neuron clusters. Panel** G** shows the UMAP of the integrated ENS meta-atlas. EMN: excitatory motor neuron; IMN: inhibitory motor neuron; IN: interneuron; IPAN: intrinsic primary afferent neuron

For data comparison and integration, we used the R package Seurat, for quality control metrics and unsupervised clustering and Seurat’s SCTransform v2 to integrate each dataset’s sequencing replicates (referred to as runs) [33–37, 39, 40]. Initially, similarities between enteric neuron scRNA-seq datasets were assessed by several standard bioinformatic approaches. All datasets were plotted concurrently using the same uniform manifold approximation projection (UMAP) parameters (Fig. 2A-F). UMAP plotting is a dimensionality reduction approach that shows how cells, represented as dots, are transcriptionally similar to each other as displayed by the distance between cells. Cells that are closer together are transcriptionally more similar than cells that are widely separated on a UMAP. These plots reveal the differences in cell numbers between datasets. Second, we evaluated expression of genes known to mark discrete enteric neuron types and displayed the expression of these marker genes for each dataset using dot plots (Fig. 2A’-F’). This approach revealed consistent detection of canonical neuron subtype markers across datasets, especially those with highest cell numbers. We also performed differential gene expression within each dataset to find putative cluster marker genes (Seurat’s FindAllMarkers), which can be found in Supplementary Tables 1–7.

#### Removal of putative non-neuronal clusters pre-integration

We then focused our meta-analysis on clusters that were present in all datasets. We removed dataset-specific clusters in the meta-atlas, as the presence of these would not aid in determining cell identity for these neuronal types because these do not benefit from additional cells counts and therefore will not gain higher resolution. Specifically, we noted that the May-Zhang 10 × data clusters 7, 11, 12, 13, and 14 (corresponding to clusters 4, 11, 12, 13, and 14 from [10] in-publication processing) were not present in other datasets, so these were removed (Supplementary Fig. 1 A) [10]. While the May-Zhang 10 × dataset's in-publication cluster 8 was an unassigned cell type, it was marked by expression of neuronal genes *Nos1*, *Gal*, and *Sst* (at lower levels), so we chose to leave these cells in the meta-atlas (Supplementary Fig. 1B) [10]. We also removed cells that concurrently expressed glial and neuronal markers, as these cells were not relevant for an enteric neuron meta-atlas. Specifically, we removed the Morarach dataset's cluster 3 and cluster 0 in the Wright et al. dataset, as these were marked by a mix of enteric glial and neuronal markers (Supplementary Fig. 1 C,D) [11, 12]. We postulate that the differences in cluster presence between datasets are attributable to either different transgenic lines, methods used to isolate cells, or slight variations in the tissue segments harvested.

### Integration of reprocessed datasets to generate a single cell transcriptomic meta-atlas of enteric neuron types

**Fig. 3:**
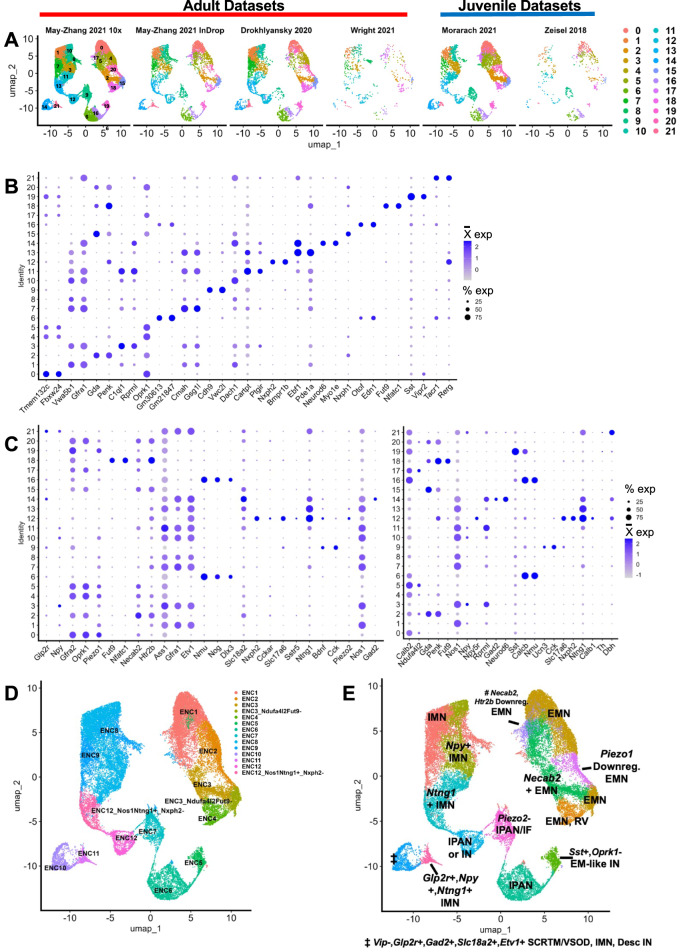
Post-integration gene expression in unsupervised clusters reveal distinct neuronal classes in relation to prior classifications. **A** UMAP from Fig. 2G grouped and split by the dataset of origin, showing the results of batch correction of the cells from each dataset. **B** Dot plot of significant differentially expressed upregulated genes per cluster based on high log2 fold change and exclusive cluster expression. **C** Dot plot shows expression of enteric neuron type marker genes sourced from Dharshika & Gulbransen Fig. 2 (Left dot plot) and Morarach and colleagues’ Fig. 1j (Right dot plot). We used the expression of these marker genes to assign each cluster a putative enteric neuron type and ENC cluster number shown in Table 2.^2,11^ **D** UMAP from Fig. 2G grouped by putative “ENC” classifiers from [11] **E** UMAP from Fig. 2G grouped by putative type corresponding to the first column in Table 2. EMN = Excitatory Motor Neuron; IMN = Inhibitory Motor Neuron; RV = Rare Variant; EM-like = Excitatory Motor-Like; IN = Interneuron; IPAN = Intrinsic Primary Afferent Neuron; IF = Intestinofugal Afferent Neuron; SCRTM = Secretomotor; VSOD = Vasodilator; Desc = Descending; Downreg. = Downregulated. A cluster of lower quality is indicated by #

To integrate all datasets into one, we integrated by biological replicate, which resulted in the integrated dataset represented by a UMAP dimensionality reduction with 22 clusters generated via unsupervised clustering (Fig. 2G). We confirmed that the characteristic neuron type marker genes initially detected in each individual dataset (Fig. 2A’-F’) were consistently observed in the integrated dataset by re-examining these markers using dot plots (Fig. 2G’). As expected, the markers are expressed in distinct patterns after integration. The process of batch correction is an analytic technique designed to remove effects in the data that are due to the methods by which the data are generated, or technical variation, allowing discernment of true similarities or differences between datasets upon integration. Batch correction can be performed by any of several approaches [41]. In our processing we applied batch correction via SCTransform v2 integration [39, 40]. To demonstrate this batch correction approach performed appropriately, we show UMAP and PCA plots with cells and split by cluster (Fig. 3A, Supplementary Fig. 1E-G). If batch correction were not successful, the plots could show one of two possibilities. Either the cells from each dataset would not be dispersed relatively evenly throughout each cluster, or clusters of differing cell types would be merged.

When combining datasets, we can achieve a depth of cell types that is greater than the individual datasets alone. For example, in our reprocessing of each individual dataset, each displayed varying numbers of neuronal types, and the datasets with the fewest cells had less diversity of neuronal types in their clustering. Interneurons and IPANs all clustered together in our reprocessing of the data from [12] (cluster 5), and interneurons marked by *Sst* and IPANs marked by *Nxph2* and *Ntng1* were low in number in [13] (clusters 8, 7; Figs. 2E’,F’) [12, 13]. In the meta-atlas dataset, these cells clustered with their appropriate counterparts from datasets with larger cell counts (Figs. 2G,G’,3A).

### Expression markers of meta-atlas neuron types detected via differential gene expression

With higher cell counts brought together by combining individual scRNA-seq datasets, one can further mine the meta-atlas for hypothesis generation and compare the integration to its original dataset components. We assessed a few basic questions to probe the integrated meta-atlas dataset. First, we aimed to identify genes that are significantly differentially upregulated in each cluster versus all other clusters, as these might assist in detection of expression markers of novel neuron types that were missed in the individual analyses. To accomplish this, we performed differential gene expression analysis for each cluster versus all others using Seurat’s FindAllMarkers function [33–37]. We identified the top differentially upregulated genes per cluster by filtering the FindAllMarkers results to those genes that had average log2 fold change of greater than 1 and a significant Bonferroni adjusted *p*-value (*P* < 0.05; Supplementary Table 8). We then visually examined where on the UMAP these genes were expressed to prioritize those whose expression was primarily within a single neuron cluster. This process produced a list of putative marker genes for each of the resulting 22 clusters (Fig. 3B).

### Meta-atlas identifies 14 commonly shared enteric neuron types based on prior literature definitions

The meta-atlas can also allow us to compare proportions of cell types within each component dataset as well as use previous definitions of enteric neuron types to determine which clusters correspond to which neuron type and identify enteric neuron types shared across datasets. We have presented these data in table format and an accompanying dot plot (Table 2; Fig. 3C). We use and add to the neuronal cell definitions from Dharshika & Gulbransen’s Fig. 2, which shows the putative anatomy and morphology of cell types marked by gene expression from scRNA-seq, as well as use the “ENC” cluster definitions from Morarach and colleagues [2, 11]. Using both ENC and Dharshika & Gulbransen’s marker gene definitions, we plotted expression of these marker genes for each cluster, showing distinct patterns (Fig. 3C; Dharshika & Gulbransen: left dot plot; Morarach “ENCs”: right dot plot). Our clusters often expressed genes corresponding to more than one annotated cell type, so we opted to list all possible cell type assignments in Table 2. Of note, Dharshika & Gulbransen use *Slc18a2* expression as a marker for enteric glia [2]. However, we found that this gene is also expressed in our neuronal cluster 14 (as well as lowly expressed in other clusters), which we posit is a secretomotor, vasodilator, inhibitory motor, or descending interneuron because of its expression of *Glp2r*, *Gfra1*, *Etv1*, and *Gad2*. [2] Therefore, *Slc18a2* may not be a reliable marker exclusively for enteric glia in situ. Integration of Table 2 and Fig. 3C suggests that 14 distinct types of enteric neurons are shared across all the datasets as annotated on the UMAP shown in Fig. 3E. All the “ENCs” are represented across the datasets, yet the higher cell count of the meta-atlas allowed detection of further putative neuronal subtypes.

**Table 2:** Distribution of cells across unsupervised clusters per dataset

**Putative Neuron Type ^**			**Cell Counts Per Dataset**						
	**Putative “ENC”** *	**Meta-Atlas Cluster**	**Adult**				**Juvenile**		**Meta-Atlas Total**
			**May-Zhang 10X**	**May-Zhang InDrop**	**Drokhlyansky**	**Wright**	**Morarach**	**Zeisel**	
Excitatory Motor	ENC1	0	2341	557	401	6	628	97	4030
Inhibitory Motor	ENC8	1	1653	334	562	59	190	24	2822
*Necab2* + Excitatory Motor	ENC3	2	917	272	231	34	771	31	2256
*Npy* + Inhibitory Motor	ENC8	3	1050	277	264	56	450	28	2125
Excitatory Motor	ENC2	4	1187	239	313	16	301	56	2112
*Necab2* + Excitatory Motor	ENC1	5	946	289	100	7	688	52	2082
Intrinsic Primary Afferent	ENC6	6	1187	195	213	8	120	45	1768
Inhibitory Motor	ENC9	7	1039	214	342	37	115	5	1752
Inhibitory Motor	ENC9	8	970	377	143	111	130	7	1738
*Piezo2*- IPAN/Intestinofugal Afferent	ENC7	9	1075	201	60	10	270	79	1695
*Npy* + Inhibitory Motor	ENC8	10	905	180	313	44	225	17	1684
*Ntng1* + Inhibitory Motor	ENC8	11	819	143	145	25	415	37	1584
IPAN/Interneuron	ENC12	12	817	209	310	50	140	21	1547
*Ntng1* + Inhibitory Motor	ENC12, *Nxph2*-	13	986	197	220	12	99	8	1522
*Vip*-, *Glp2r* + , *Gad2* + , *Slc18a2* + , *Etv1* + Neurons (Secretomotor/Vasodilator, Inhibitory Motor, Descending Interneuron)	ENC10	14	859	166	159	9	234	10	1437
Excitatory Motor	ENC3, *Ndufa4l2*, *Fut9***-**	15	526	144	253	18	223	17	1181
Intrinsic Primary Afferent	ENC6	16	725	97	146	14	116	52	1150
*# Necab2*, *Htr2b* Downregulated, Excitatory Motor	ENC1	17	261	175	71	60	531	40	1138
Excitatory Motor, Rare Variant	ENC4	18	482	127	264	42	135	26	1076
*Sst* + , Oprk1- Excitatory-like Interneuron	ENC5	19	545	113	200	6	106	8	978
*Piezo1* Downregulated, Excitatory Motor	ENC2	20	512	104	118	12	201	11	958
*Glp2r* + , *Npy* + , *Ntng1* +, Inhibitory Motor	ENC11	21	243	77	159	13	49	3	544

Table of putative neuron cell counts grouped by cluster and dataset of origin

**^**Putative neuron types are derived from Dharshika & Gulbransen Fig. 2

^2^ *ENC designations are based on prior marker gene classifications from Morarach and colleagues

^11^ A cluster of lower quality is indicated by #

### Differential abundance of putative enteric neuron types detected by age and intestinal segment

**Fig. 4:**
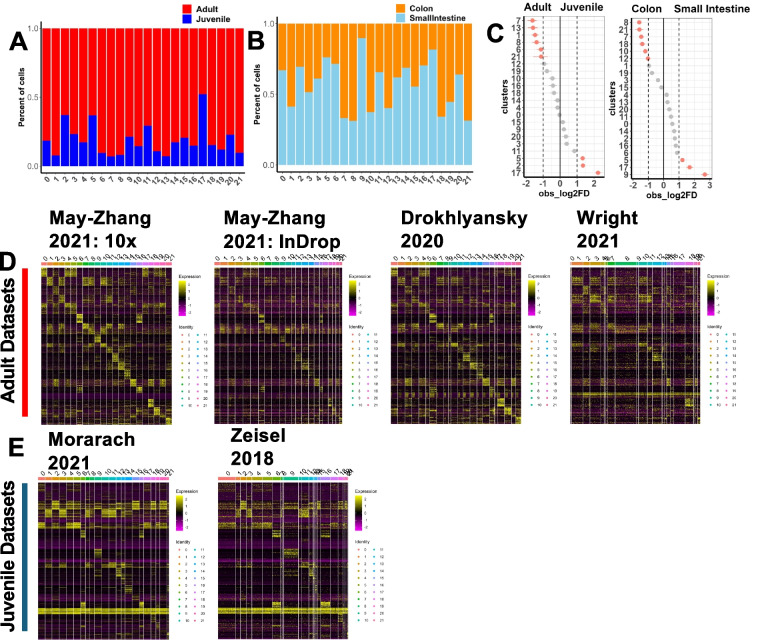
Post-integration cell distribution in unsupervised clusters, marker genes uncovers similarities and differences between datasets. **A** Stacked bar plot displays the distribution of adult and juvenile cells per cluster in the meta-atlas scRNA-seq dataset. **B** Stacked bar plot displays the distribution of colon and small intestine cells per cluster. **C** Permutation analysis displayed on a forest plot comparing the abundance of adult and juvenile cells per cluster and abundance of colonic and small intestine cells per cluster. **D** Heatmap displaying expression for the top 30 genes for each cluster from FindAllMarkers differential expression results of the meta-atlas dataset ranked by lowest *p*-value and highest log2 fold change plotted on the four adult datasets. **E** Heatmaps displaying the same 30 genes in **F** plotted on the two juvenile datasets

Integrating these datasets gives us an opportunity to identify distinctions between datasets and various annotations such as age and tissue section. To understand the differences in clustering distribution across age and tissue type (Fig. 4A,B, Table 2), we performed Monte-Carlo permutation tests for each cluster (Fig. 4C) [42]. We found that clusters 7, 13, 1, and 8 had significant proportional bias towards adult ([10], [16], and [12]) cells while clusters 5, 2, and 17 were proportionally biased towards juvenile cells ([11],[13]; Fig. 4C, left). We also found that at least clusters 21, 8, and 7 had proportional bias towards colonic cells while clusters 5, 7, and 9 were proportionally biased towards small intestinal cells (Fig. 4C, right). These patterns hint at how enteric neuron populations change over time and across intestinal regions.

### Distinct expression patterns of top neuronal marker genes detected by age in the meta-atlas

**Fig. 5:**
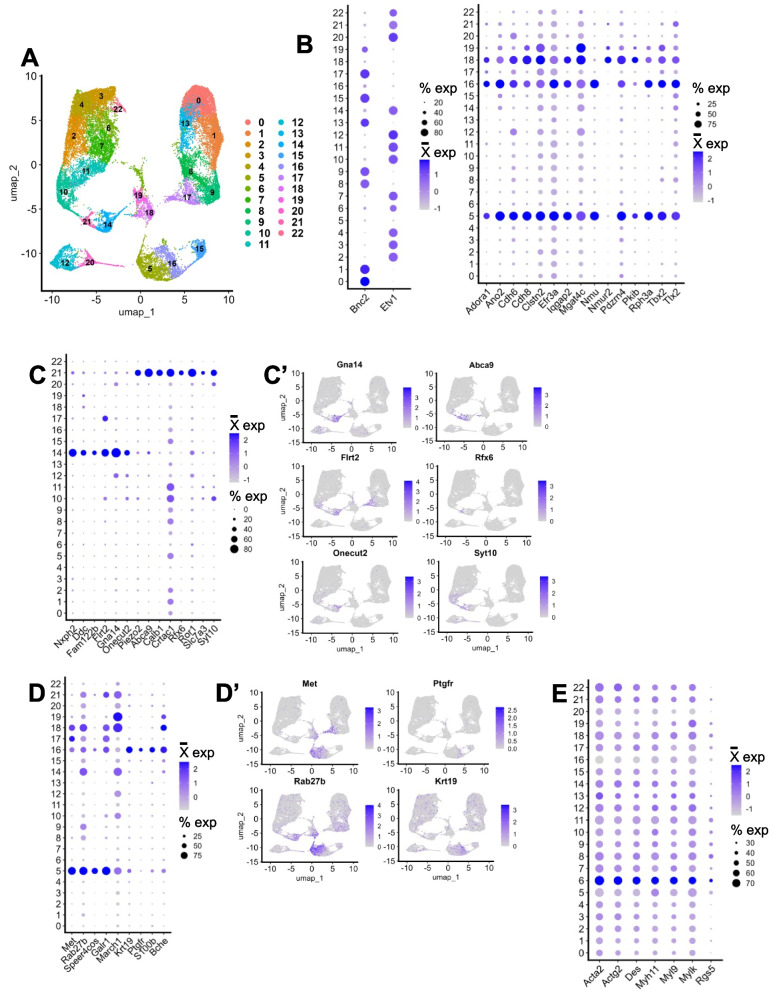
Probing the adult ENS meta-atlas for subclasses of enteric neurons. **A** UMAP of the new adult-only integrated meta-atlas. **B** Dot plots showing either Bnc2 and Etv1 expression for all clusters (left) except, 18, 16, and 5, which have distinct markers (right). **C** Dot plot showing expression of differentially expressed genes between clusters 14 and 21, putative subclusters of ENC12, with three representative genes’ expression per cluster plotted on the UMAPs in **C**’. **D** Dot plot showing expression of genes that typically mark non-neuronal types are expressed at low levels throughout the data but are most prominent in cluster 23. **E** Dot plot showing expression of differentially expressed genes between clusters 5 and 16, putative subclusters of ENC6, with two of these representative genes’ expression per cluster plotted on the UMAPs in **E**’

To identify differences in expression between datasets, we used our top 30 genes per cluster from the FindAllMarkers results of the integrated dataset to plot heatmaps displaying expression patterns (Fig. 4D,E, Supplementary Table 8). When we plot the gene expression for the subset adult datasets, the patterns of markers per cluster are relatively maintained, with dataset from [12] displaying the least consistent pattern (Fig. 4D). However, when plotting the subset juvenile [11] and [13] datasets, many clusters do not have their own patterns of gene expression (Fig. 4E). This leads us to conclude that the adult datasets, especially [10] 10X dataset with its greater cell count, bias the FindAllMarkers differential gene expression data. We surmise that any further conclusions about cell type diversity must come from either the adult or juvenile, and not both. Although explicit comparisons of the two stages could be informative for finding commonalities across ages. As a result, we decided to reintegrate only the adult datasets and use these data for downstream analyses (Fig. 5A). We also perform FindAllMarkers differential gene expression analysis on these adult integrated data (Supplementary Table 9).

### Expression markers of clusters that do not express enteric neuron lineage markers *Bnc2* and *Etv1*

With the effectively greater-per-cluster cell numbers, we were able to assess potential novel gene expression markers. For example, Morarach and colleagues found that the two divergent neuronal trajectories in the developing ENS were marked by the genes *Bnc2* and *Etv1,* respectively and the expression of these genes carried into juvenile stages [11]. However, in that study (refer to Figs. 2e, 6f in [11]), it appears there are cells that do not express *Bnc2* (ENC6, some of ENC7, end of Branch B) in the same way that the other branched linage expresses *Etv1* almost entirely [11]. Following integration of the adult ENS single cell datasets, the distinct expression of *Bnc2* and *Etv1* is apparent (Fig. 5B, left). This distinction provides the opportunity to probe the adult meta-atlas at higher resolution than was done in prior individual datasets to determine whether cells that lack *Bnc2* or *Etv1* express a gene or set of genes that distinguish these separate populations that might be expressed at fetal stages and maintained into adulthood. To explore this possibility, we took all our integrated clusters that expressed either *Bnc2* or *Etv1* and performed differential gene expression versus the clusters that do not appreciably express either of these two genes (Supplementary Table 10). At least 13 prominent genes emerged from this process (Fig. 5B, right), each of which mark the clusters that do not express *Bnc2* or *Etv1*. For example, *Tbx2* has been implicated in enteric neuron subclasses based on differential expression between neuronal types via immunohistochemistry and prominent expression among cholinergic enteric neurons although loss of this gene goes not alter density of *Chat* + enteric neurons [12]. Postnatal motility experiments in adult or juvenile *Tbx2*^*−/−*^ mice were not possible because *Tbx2* mutants die shortly after birth [12]. Another gene differentially expressed in these clusters, *Tlx2,* has been shown to interact with *Phox2a/b* in neural crest-derived cell development, with three *Tlx2* knockout mouse models exhibiting ENS defects [43–47]. *Iqgap2* has not been implicated in motility studies to date, although it has been reported to be required for inflammatory responses in the mouse colon [48]. Identification of these genes as markers that distinguish these neurons from those expressing *Bnc2*/*Etv1* offers the opportunity to interrogate these cell types further in adult and juvenile stages.

**Fig. 6:**
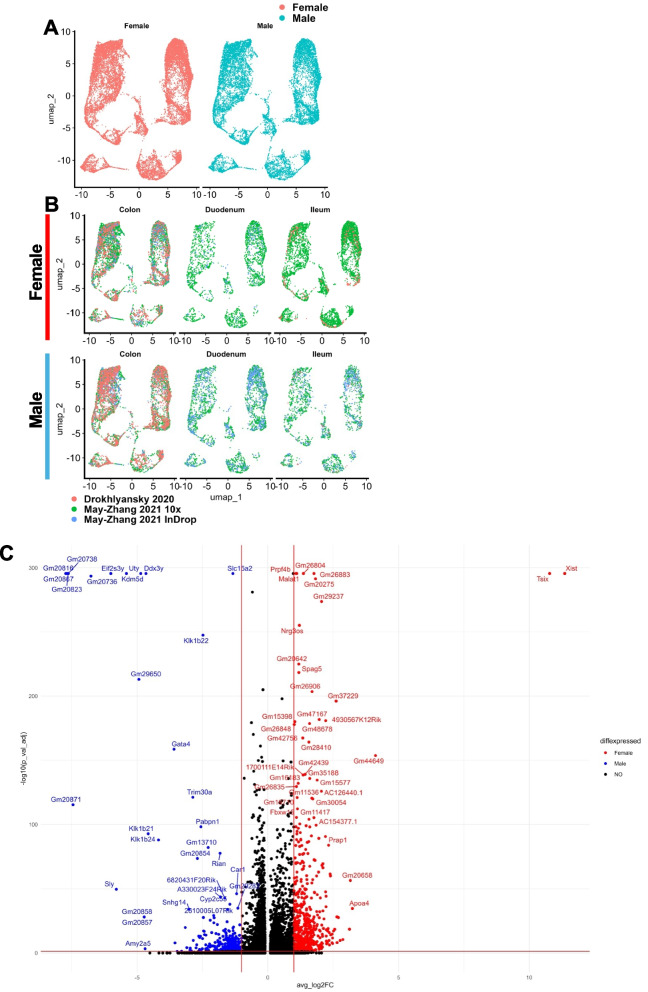
Sex and dataset differences identified in the adult meta-atlas. **A** Distribution of sex in the dataset visualized through a split UMAP. **B** UMAP displaying distribution of datasets of origin in the adult meta-atlas split by intestinal segment and stacked by sex. **C** Volcano plotting differential gene expression in all cells between male (blue) and female (red). Differentially expressed genes with *P* = 0 were adjusted to *P* = 3.070490e-296 for purposes of visualization

### Differential gene expression analysis identifies differences between *Nxph2* + ENC12 and *Nmu* + ENC6 subclusters

Increasing cell numbers through data integration also allows further subdivision of clusters to resolve previously unappreciated neuron subtypes. In prior reports excitatory neurons expressing *Nxph2*, *Sstr5*, and *Piezo2* (ENC12) were reported as a complex cluster both in scRNA-seq profile and in morphology observed by immunohistochemistry [11]. Subtypes of this cluster were evident in UMAP analysis from Morarach and colleagues, although cell numbers were fewer than other clusters in those datasets [11]. We probed the adult integrated meta-atlas dataset to determine whether any of these subtypes could be further discerned. In the adult meta-atlas, clusters 14 and 21 coincided with Morarach ENC12 as verified by expression of ENC12 marker gene *Nxph2* and ENC12 subtype marker *Piezo2*, respectively (Fig. 5C). Utilizing the higher adult meta-atlas cell count for ENC12 and Seurat’s FindMarkers differential gene expression analysis, we identified additional adult marker genes for these subclusters, including *Abca9* and *Syt10* for cluster 21 and *Flrt2* and *Gna14* for cluster 14 (Supplementary Table 11, Fig. 5C,C’).

Another example of greater cluster subdivision gained from the meta-atlas was noted for the *Nmu* + cluster, where *Nmu* expression has been shown to mark IPANs [11]. When performing unsupervised clustering in both the complete and adult-only meta-atlas, we noticed that the *Nmu* + cluster was split into two subclusters (Figs. 2G, 3A, 5A cluster 5 versus cluster 16). To determine whether this clustering could be reflective of biology and additional enteric neuronal subtypes, we performed differential gene expression between the adult meta-atlas clusters 5 and 16 (Supplementary Table 12). By visual inspection of the top differentially expressed genes per cluster for specificity, we found at least 8 differentially expressed genes that may be tested for functional relevance (Fig. 5D,D’). Two of these genes, *Ptgfr* and keratin gene *Krt19*, distinguish cluster 16 (Fig. 5D,D’). While Hepatocyte growth factor (HGF) receptor *Met* and the previously identified gene *Rab27b* mark cluster 5 (Fig. 5D,D’) [10].

### Non-neuronal expression markers persist in the adult meta-atlas

When integrating datasets from different sources, clusters that might not have been previously identified could manifest. In the complete integrated and adult meta-atlas datasets, we observed that meta-atlas cluster 8 and adult meta-atlas cluster 6 express genes that typically mark non-neuronal cell types (*Acta2*, *Actg2*, *Des*, *Myh11*, *Myl9*, *Mylk*, *Rgs5*; Figs. 2G, 5E, Supplementary Fig. 2 A). When split by dataset of origin, the adult integrated dataset UMAP shows that most of these cells come from the May-Zhang et al 2021 datasets (Supplementary Fig. 2B) [10]. When these non-neuronal marker genes are mapped back to the original May-Zhang datasets [10], we find that they are widely expressed throughout, with highest expression in the unclassified clusters (Fig. 2A,B, Supplementary Fig. 2 C,D). These genes were also expressed in the other two datasets. In the reprocessed Morarach dataset, there is limited expression of these non-neuronal genes in cluster 3 (Fig. 2C, Supplementary Fig. 2E). Expression is more dispersed in the reprocessed Drokhlyansky dataset, with highest expression in clusters 1, 6, and 7 (Fig. 2D, Supplementary Fig. 2 F). It’s possible this dispersed expression of non-neuronal genes could be the result of ambient RNA due to muscle cell lysis during generation of nuclei from laminar muscle preparations. This expression could also arise from hetero-doublets (two nuclei of distinct cell types in the same droplet), although this is less likely given the use of FACS-sorting with size gating. However, Zeisel and colleagues reported the presence of enteric mesothelial fibroblasts marked by expression of *Pth1r*, *Kcnj8*, *Abcc9*, *Cd82*, *Tagln*, *Dcn*, *Lum*, *Pdgfra*, *Sox10*, *Aldh1a3*, and *Anxa11 *[13]. In the integrated adult meta-atlas, a subset of these genes (*Kcnj8*, *Abcc9*, *Cd82*, *Tagln*, *Dcn*, *Lum*, and *Pdgfra*) exhibit expression mostly in cluster 6 (Fig. 5A, Supplementary Fig. 2G). Whether the differences between datasets are due to technical challenges in sample processing or reflect biologically relevant cell types or states remains to be determined.

### Sex differences identified through differential gene expression

Most of the publications used to source data for the integrations of meta-atlas used recorded or estimated sex to either display cell distribution by sex, account for sex in differential gene expression (DGE) analysis, or perform DGE analysis by this recorded or estimated sex metadata. Because the aggregate higher cell quantities exceeded those of each individual dataset, we used the opportunity to probe the meta-atlas for sex differences via DGE in our adult integrated data. However, since Wright and colleagues did not record sex, and since the contributions to the overall data are smaller than the other three, we elected to remove the Wright et al. scRNA-seq runs for the sex-differences analysis [12]. In order to track sex, we used both the annotations from the Drokhlyansky and May-Zhang publications, as well as the expression of known genes *Ddx3y*, *Uty*, *Tsix*, and *Xist* (Supplementary Fig. 3 A,B,C,D,E,F,G,H) [6, 10]. Before going forward with the DGE analysis, we examined proportions of each dataset’s cells per sex per tissue segment. The Drokhlyansky et al. dataset had zero cells in duodenum for both sexes and only 63 cells in male ileum versus 408 cells in female ileum, while May-Zhang et al.’s InDrop data had no female cells in ileum versus 571 male cells and 1000 male cells versus 233 female cells in duodenum (Fig. 6A, B). In addition, May-Zhang et al.’s 10 × dataset had much more female cells in ileum than in male (5664 versus 1562; Fig. 6A,B). Because of these skews, performing DGE by sex per tissue may uncover dataset-specific gene expression instead. Therefore, we opted to perform sex-specific differential gene expression analysis with all tissues included using logistic regression in an attempt to regress out dataset-specific effects (Fig. 6C, Supplementary Table 13). For the top differentially expressed genes labeled in the volcano plot, we examined expression split by sex and grouped by both dataset of origin and tissue segment to determine if expression stratified by sex was consistent across these variables (Supplementary Fig. 4). As expected, *Ddx3y*, *Eif2s3y*, *Uty*, and *Kdm5d* exhibited male-specific expression and *Tsix* and *Xist* were female-specific (Supplementary Fig. 4 A). However, several genes with the prefix “*Gm-*” were dataset-specific, including several in the Drokhlyansky colon dataset and several in May-Zhang 10× dataset (Supplementary Fig. 4B). Sex-biased expression was most consistent across dataset and tissue segment for *Slc15a2* and *Malat1* and was less consistent for *Klk1b22*, *Prpf4b*, and *Nrg3os*, which were expressed at lower levels in the May-Zhang InDrop dataset (Supplementary Fig. 4 C). Together, these data show that while sex-specific expression can be found in the adult meta-atlas, these results can be skewed by uneven distributions of clusters in the UMAP across tissue segment and sex as well as dataset-specific genes.

## Discussion

Here we have illustrated how the integration of six individual scRNA-seq or snRNA-seq datasets provides higher power for statistical analyses, while also adding more complexity. Meta-analysis of juvenile to adult mouse myenteric enteric neuron scRNA-seq datasets produced higher cell numbers for cell types shared across datasets, enabling verification of prior enteric neuron classifications, extending the identification of additional marker genes, and offering greater resolution for the subdivision of clusters. Our approach also allowed us to identify differences between datasets including major expression differences across age, differences in cell type distribution, and identification of clusters and cell populations that are specifically enriched within individual datasets or intestinal segments. In our permutation analyses, we found that a group of putative ENC12-like, *Ntng1* + inhibitory motor (cluster 13), ENC8-like inhibitory motor (cluster 1), and ENC9-like, inhibitory motor (clusters 7, 8) neuron types were more prevalent in adult. While ENC1 and 3-like, *Necab2* + excitatory motor (clusters 2, 5) and ENC1-like, *Necab* and *Htr2b* downregulated excitatory motor neuron types were more abundant in juvenile ages (Figs. 3D, 4C, Table 2). In addition, we found that putative inhibitory motor neurons (clusters 21, 8, 7, 10), a rare variant of excitatory motor (clusters 18), and an IPAN or interneuron type (cluster 12) are more prevalent in the colon. Finally, we found that inhibitory motor (clusters 5, 17) and a *Piezo2*-, ENC7-like neuronal types are more prevalent in the small intestine. These findings point to specific genes that mark enteric neuron clusters that our analysis suggests may change in composition over time and differ in abundance between intestinal regions. Future experimental validation using lineage tracing and in situ studies are needed to document these differences and demonstrate the value of these genes for distinguishing enteric neuron subclasses. There was some overlap observed between clusters that significantly differ between age and intestine segment, which may indicate dataset-specific effects in the permutation analysis. Our attempt to identify sex-specific differential gene expression analysis confirmed previous findings. However, dataset-specific effects can complicate this analysis, such as higher cell abundance for a specific intestinal segment within a dataset, even after regressing out dataset metadata.

In our analysis of the adult meta-atlas, we discovered gene expression markers of subclusters for both *Nxph2* + which marks excitatory neurons, “ENC12”, and *Nmu* + which marks IPANs, “ENC6” as initially described by Morarach and colleagues. In addition to previously identified *Piezo2* and *Nxph2*, we extended the known marker genes for “ENC12” subcluster 14, with differential expression of *Gna14*, *Flrt2*, and *Onecut2*, while subcluster 21 expresses *Abca9*, *Rfx6*, and *Syt10* when these subclusters are compared (Figs. 5C,C’). *Flrt2* has novel tumor suppressor activity in breast cancer and localizes to pre- and post-synapses in the postnatal developing hippocampus where it may play a role in synapse formation [49, 50]. *Gna14* is downregulated in *Pou3f3* + colonic versus *Pou3f3*- noncolonic immature enteric neurons at 17.5 dpc, suggesting higher expression in noncolonic enteric neurons [12]. While *Gna14* was previously reported as a marker gene for ENC12 enteric neuron cluster, our analysis suggests it actually marks a subset of this ENC12 population seen as cluster 14 in the meta-atlas [11, 12]. *Abca9*, along with other *Abca*-family genes, was recently identified as differentially expressed in superior frontal cortex and cerebellum of a younger-onset form of dementia when compared to controls [51]. These changes in *Abca* gene expression were correlated with neuronal types related to inflammation [51]. Identification of these additional genes for further subdividing enteric neuron clusters offers an exciting opportunity for their future use in vivo to distinguish and investigate enteric neuron subclasses.

The power of the meta-atlas for further resolution of subclusters again becomes evident when examining the *Nmu* + clusters 5 and 16 in the meta-atlas, previously labeled as “ENC6” [11]. Prior analysis probing *Nmu* + ENC6 cells for subclusters in the analysis by May-Zhang and colleagues across the intestinal segments did not split this cell population via unsupervised clustering of a single dataset (May-Zhang et al., 2021 Sup. Figure 5A) [10]. In contrast, these subclusters split in the adult meta-atlas via unsupervised clustering (Fig. 5A, clusters 5 and 16). By performing differential expression analysis between these *Nmu* + clusters, we found that subcluster 5 differentially expressed *Met* and *Rab27b* (Fig. 5D,D’). Interestingly, we noted that the hepatocyte growth factor (HGF) receptor *Met* marks *Nmu* + cluster 5, as well as clusters 17 and 18*. Met*, which is important for development of a subset of IPANs that regulate motility and injury response pathways, exhibits differential expression for cluster 5 (Fig. 5D,D’) [52]. *Met* has also recently been suggested as a marker of a putative mesoderm-derived enteric neuron lineage in the aged intestine. However, further validation using fluorescence-activated cell sorting to enrich these putative Met + populations is needed [16]. *Nmu* + cluster 5 also differentially expressed higher levels of *Rab27b*, which was also found to be mostly restricted to a subset of *Nmu* + neuronal cells in the integrated findings from the May-Zhang 2021 study (See May-Zhang et al., 2021 Sup. Figure 5A; Fig. 5D,D’) [10]. We also found that *Krt19*, a keratin gene, was upregulated and selectively expressed in *Nmu* + subcluster 16. We confirmed that expression of *Krt19* was also enriched in cluster ENT9 in the juvenile dataset from the Zeisel 2018 study (not explicitly in the publication, although present on MouseBrain.org), which is an *Nmu* + cluster (Fig. 5D,D’) [13, 53]. Finally, *Ptgfr* was found to be differentially upregulated in *Nmu* + cluster 16 and is expressed mainly in a subportion of this cluster (Fig. 5D,D’). In the individual Morarach dataset *Ptgfr* is observed in clusters ENC5 and ENC6 and appears to be restricted to this *Nmu* + subcluster in later adult stages (see Morarach et al., 2021 Fig. 2a, left) [11]. Like the subclustering described above for “ENC12”, these findings must be validated with in situ labeling, analysis of cell morphology, or flow sort purification to establish these genes as markers of genuine enteric neuron subtypes. Given the rarity of these neuron subclusters parsed by the additional marker genes, future validation efforts will need to incorporate significant enrichment strategies or rely on recombination-based cre tracing to derive experimental evidence that these subclusters reflect authentic enteric neuron subtypes within the adult ENC6 and ENC12 populations.

The potential applications of an enteric neuron meta-atlas are tremendous, as this framework can be used to assess changes in enteric neuron profiles resulting from age, disease, diet, or microbiome. Multiple groups have posted individual datasets online for continued access [5–19]. However, to date no single repository exists for aggregated ENS data that is designed to facilitate access by investigators without bioinformatics expertise. To encourage further efforts and begin to address this challenge for the field, we offer the R objects and code from the present meta-atlas compilation on Zenodo so other investigators can readily extend and mine this meta-atlas going forward.

Our meta-atlas relied upon robust batch correction approaches to reduce variation between datasets as is typically done. However, recent advances in barcoding and multiplexing strategies position the field for simultaneous sequencing of distinct samples that will facilitate comparative analysis and further reduce variation [54, 55]. Multiplexing would be particularly useful for analysis of ENS change across the lifespan as iterative sampling for a single mouse inbred strain could be readily performed by a single laboratory. Challenges will remain for controlling variance between studies due to differences in transgenic lines, genetic background of strains, diet, or times of tissue harvest due to circadian rhythms. Reporting specifics of these variables in each publication is essential so that these factors can be accounted for and noted when differences are observed between datasets. Variation among human studies will remain an even greater challenge compared to the ability to control environment, including microbiome, in rodents.

Choosing to process these data in readily available, previously aligned forms, has benefits and detriments in a meta-analysis such as this. For example, if one does not have access to enough computational resources to perform alignment via a program such as CellRanger, then individual groups may not be able to process the data from FASTQ files onwards. By contrast, it is relatively easy to download processed data files and replicate the data display to observe how it matches the results from each publication. However, differences in alignment approaches, mapping references, or other data processing methods from each publication can lead to complications, such as was the case for one of the InDrop datasets from the May-Zhang study [10]. Reprocessing and realigning the FASTQ files could potentially offer cleaner results across all datasets, due to greater uniformity of gene symbols used for combining the counts for each gene across all datasets during the merging process. If authors consistently made available raw and filtered gene expression matrices, it would be easier to investigate clusters origins and distinguish true biological variation versus technical differences. Otherwise, gene synonyms and alternative aliases across different mapping references can prevent counts from being properly binned in the resulting matrix.

Importantly, the field still faces the challenge of profiling young, healthy adult human enteric neurons at single cell resolution. The prior May-Zhang study relied on laser capture to gather entire ganglia sections from young adults [10]. Drokhlyansky and colleagues used MIRACL-seq to assess RNA from older colorectal cancer patients [6]. Because enteric neurons are affected by colorectal cancer, isolation of single enteric neurons for transcriptional profiling from young healthy adults is still needed [56, 57]. While optimized methods of tissue dissociation that maintain neuronal viability would enable this process, use of frozen tissue isolates to generate nuclei, combined with sequencing of vast cell numbers as sequencing costs decline, will likely succeed in circumventing this issue [58].

A greater challenge for the ENS field will be linking the emerging transcriptional profiles of cell types with the historical morphological and electrophysiological data that has previously been the gold standard for classifying enteric neurons. Expression of single immunohistochemical markers has in some cases facilitated linking a transcriptionally defined neuron cluster with prior knowledge of neuron classes [11]. May-Zhang and colleagues relied upon expression of known markers detected within some neuron types to propose the neuronal identity of clusters in their single cell data [10]. Linking gene expression to cell morphology can be achieved through analysis of cell shape labeled by fluorescent reporters and localizing neuronal processes [11, 19]. Relating transcriptional profiles to electrophysiological data is more challenging. One approach could be to sequence single neurons after electrophysiological studies as has been done for brain neurons [59]. However, culture of enteric neurons to evaluate their polarization profiles may alter gene expression patterns. As sequencing technologies advance, it is more likely that spatial sequencing or transcriptional profiling of live cells will lead to success in defining the transcriptome of healthy human enteric neurons in situ [60].

The ENS field has not yet come to agreement on nomenclature of enteric neuron types. Each source dataset in this meta-atlas utilizes its own nomenclature and has differing numbers of clusters and types. The most easily followed nomenclature is to designate clusters by their most prominent and specifically expressed genes. Although, to date many groups reference the Enteric Neuron Cell (ENC) cluster designations used by Morarach and colleagues, which we frequently reference here. In a meta-atlas of lung, consensus cell type nomenclature was harmonized between datasets via a hierarchical reference framework that mapped cell identities across each of its 166 samples building from dataset-specific cell type labels [61]. While this approach was complex due to the large number of samples and the number of cell types in lung tissue generally, a similar approach could be taken to describe enteric neuron types as more scRNA-seq data is produced. By linking this approach with morphological and physiological definitions of enteric neurons, the ENS field has the potential to arrive at consensus nomenclature on enteric neuron subtypes.

The new subclasses of enteric neurons indicated by our analysis will require confirmation through in situ or in vivo experimentation to confirm the cell subtypes revealed in this meta-atlas reflect authentic enteric neuron subtypes. Given the low frequency of these new subtypes and lack of regional location within the small intestine and colon, this will likely require great effort. However, these new classes may exert unique effects on GI physiology and we encourage those in ENS neurobiology to further investigate these novel neuronal subclasses to gain further insights for the field and determine the functionality of these cell types.

Finally, while there is great excitement given the advances in technology for both producing and analyzing scRNA-seq, we acknowledge other processes beyond transcription are important for cell identity. Translation, post-translational modifications, and protein turnover may all be at work in producing the final identity and functionality of enteric neurons. Integration of transcriptional profiles, multiplex immunolabeling, proteomics, and lipidomics will aid in realizing the full diversity of neurons within the ENS.

## Conclusions

In this work, we performed a synthesis of publications assessing the enteric nervous system at a single cell level. We shared key insights from an integrated mouse meta-atlas of both adult and juvenile and adult single cell and nucleus RNA-seq enteric neurons. In our atlas, all previously annotated enteric neuronal types are present, and several clusters were further subdivided using known and newly identified marker genes. We also identified dataset differences, sex differences, and age differences in our enteric neuron meta-atlas. These findings have great potential to improve our understanding of the ENS, following validation by the ENS research community.

## Methods

We briefly review ENS publications that utilize scRNA-seq or snRNA-seq. We then highlight how integration of scRNA-seq datasets can contribute to the field. We subsequently extract the relevant publicly available mouse enteric neuron scRNA-seq or snRNA-seq datasets assayed from the myenteric plexus, reprocess, integrate, and perform analyses to deepen transcriptional definitions of enteric neuron subtypes.

### Literature review

We collected relevant publications of which we were aware at the time of submission and used those as a starting point for review. We further utilized Google Scholar, PubMed, and the Vanderbilt University Library website interface to search for and access other papers using various related search terms, including “ENS”, “Enteric Nervous System”, “scRNA-seq”, “single cell”, and “single cell RNA-sequencing”. We then screened papers for scRNA-seq datasets and excluded papers that were not peer-reviewed (e.g. preprint servers).

### Data access and downloading ENS scRNA-seq datasets

We downloaded the data from the following sources. Wright and colleagues’ 47 to 52 days old mouse distal colon myenteric plexus snRNA-seq data was downloaded from Gene Expression Omnibus accession number GSE156905 in the form of matrix, barcode, and feature (gene) files [12]. Morarach and colleagues’ P21 juvenile scRNA-seq mouse data were downloaded from Gene Expression Omnibus accession number GSE149524 in the form of matrix, barcode, and feature (gene) files [11]. May-Zhang and colleagues’ 6-week-old mouse enteric neuron snRNA-seq data was downloaded from Gene Expression Omnibus accession number GSE153202 in the form of matrix, barcode, and feature (gene) files [10]. We downloaded Zeisel and colleagues’ processed ENS P19, P20, and P21 scRNA-seq data in the form of a.Loom file from mousebrain.org [13, 38]. Drokhlyansky and colleagues’ 11 to 104-weeks-old mouse enteric neuron data were downloaded (each cell type downloaded separately) from the Single Cell Portal at the Broad Institute website at accession number SCP1038), where the data were in the form of matrix, barcode, and feature (gene) files [6].

### Dataset reprocessing

Each dataset’s individual sc/snRNA-seq library was read into R using Seurat’s Read10x and CreateSeuratObject functions with a minimum of 3 cells and minimum of 200 features [33–37]. We then used Seurat’s PercentageFeatureSet function to get the percentage of mitochondrial gene expression per cell based on the gene prefix pattern “^mt- “. We then plotted histograms, violin plots, and FeatureScatter (Seurat plotting function) plots of percent mitochondrial gene expression, nFeature_RNA, and nCount_RNA. These were used in choosing quality control (QC) metrics when filtering the data. We also considered the QC metrics and clustering parameters listed in the publications. Please see the code for each dataset reprocessing for the specific metrics, which ranged from 200–2000 genes per cell/nuclei as the lower bound (nFeature_RNA > 200–2000). We decided that 20 percent mitochondrial RNA per cell in each dataset was of sufficient quality (percent.mt < 20). We note that the RAISIN-seq dataset from Drokhlyansky et al. have extremely high values of nCount_RNA at a mean of just under 1,000,000 [6]. This may have to do with the method (SMART-Seq2) that was used for assaying these “RAISINs” [6, 62]. After QC filtering, we applied Seurat’s SCTransform v2 to each run, regressing out percent mitochondrial genes [39, 40]. Next, we ran principal component analysis (PCA), uniform manifold approximation projection (UMAP) dimensionality reduction, Seurat’s FindNeighbors, and FindClusters. After finding clusters and visualizing the UMAP through Seurat’s DimPlot function, we used DoubletFinder to identify and filter putative heterotypic doublets [63]. This requires an expected percentage of doublets, which can be estimated based on the number of cells loaded into a 10X Chip [32]. We used the number of cells in the object to estimate the cells recovered along with a linear function based on data found on the 10X Genomics website to estimate the multiplet rate [64]. This rate was used in the round function of DoubletFinder. SCTransform v2, RunPCA, RunUMAP, FindNeighbors, and FindClusters were then run again on the data filtered for putative heterotypic doublets. After running each run of the datasets through this pipeline, we then integrated the runs using SCTransform v2 [40]. We adjusted the clustering resolution in each dataset (Fig. 2A-F) to match numbers of clusters in each original publication. See the Zenodo repository (10.5281/zenodo.17420912) for more details, where we outline code for the estimated doublet rate linear function and provide RMD files for each dataset. Code for reducing the cluster number for most datasets is in a separate R code file.

### Integration of datasets and meta-atlas analysis

First, metadata were added to each dataset object generated, including the dataset first author last name, year of publication, age mouse, tissue section, etc. After, datasets were integrated and batch corrected via Seurat V5 by sequencing run into a meta-atlas using SCTransform v2 per the Seurat tutorial (https://satijalab.org/seurat/articles/seurat5_integration) [33–37, 39, 40]. We integrated by run to control for both the tissue segment and the age of the mice at the time of neuron isolation. After integration and batch correction via SCTransform v2, we processed the meta-atlas dataset with RunPCA, RunUMAP, FindNeighbors, and FindClusters. We noticed that the May-Zhang et al., 2021 InDrop samples from run 3316 displayed gene expression consistent with contamination and removed these from the integrated dataset. Next, we normalized and scaled the “RNA” assay using Seurat’s NormalizeData and ScaleData functions. We used the PrepSCTFindMarkers function to prepare the data for differential gene expression for each cluster (using Seurat’s FindAllMarkers function). We then ran FindAllMarkers with both the SCT-corrected data and the normalized RNA data, for which the differences were minimal. We used the RNA-assay-based FindAllMarkers results going forward. We then displayed the top 30 putative marker genes per cluster (sorted by Log2FoldChange and significant Bonferroni-adjusted *p*-value from FindAllMarkers Wilcoxon rank-sum test) in a heatmap to see clustering patterns via gene expression. To reduce the occurrence of multiple clusters sharing similar expression patterns, we ran the clustering algorithm again with a lower resolution. After re-running FindAllMarkers and re-generating the heatmap, the top 30 enriched genes for each cluster showed much less overlap than was originally observed. Top cluster markers unique to each cluster were found via dplyr functions group_by(cluster) and distinct(gene) followed by visual expression confirmation that these overlapped with their corresponding clusters. We then visually examined plots generated from the top 30 FindAllMarkers results for each cluster and chose genes that were unique to each cluster.

Because of the lack of specific expression of putative marker genes in the meta-atlas clusters for the subset juvenile datasets, we opted to reintegrate the data using the same approach outlined above including only the adult datasets. The adult meta-atlas only included runs from the May-Zhang study, the Drokhlyansky study, and the Wright study [6, 10, 12].

A prior report stated that there was independent expression of *Etv1* and *Bnc2* in either of two developing “branched” neuronal populations that is maintained into juvenile stages. However, we noted that some clusters in juvenile scRNA-seq data do not express either of these markers [11]. To perform differential gene expression for *Etv1* and *Bnc2* expressing clusters versus clusters that do not express these genes, as well as finding genes that mark new subclusters, we performed differential gene expression via Seurat’s FindMarkers (Wilcoxon rank-sum test) function for these two groups. The top genes (sorted by Log_2_FoldChange and significant Bonferroni-adjusted *p*-value) were then assessed for specificity. We used the same approach when performing differential gene expression between subtypes of enteric neurons based on unsupervised clustering. The marker genes identified in the figures were found by visual inspection for exclusivity for a specific cluster across all datasets via Seurat’s FeaturePlots. We used the same approach when performing FindMarkers between cell subtypes, which is outlined further below. These were used for Figs. 5C’ and E’.

To perform differential gene expression analysis by sex, we subset the adult data further to exclude [12] because it lacked sex annotations and was pooled by sex [11]. We performed this analysis by cluster using Seurat’s Logistic Regression method, regressing out the dataset label metadata to account for dataset differences.

### Availability of data and materials

The R code used to produce the Meta-Atlas in.R format, our reprocessing of each dataset used in the construction of the Meta-Atlas, the Meta-Atlas consisting of the adult + juvenile datasets, and the adult-only Meta-Atlas dataset are available as Seurat Objects for download on Zenodo at 10.5281/zenodo.17420912.

Original datasets for the meta-atlas are accessible as follows: Zeisel 2018 dataset (https://mousebrain.org/adolescent/tissues.html under “Enteric”); Drokhlyansky 2020 datasets (https://singlecell.broadinstitute.org/single_cell under accession number SCP1038); May-Zhang 2021 datasets (Gene Expression Omnibus under accession number GSE153202); Morarach et al., 2021 (Gene Expression Omnibus under accession number GSE149524); and Wright et al 2021 datasets (Gene Expression Omnibus under accession number GSE156905).

## Supplementary Information

Supplementary Material 1. Supplementary Figure 1. Identification of putative non-neuronal clusters to remove for effective meta-atlas generation and batch correction. A-C Dot plots displaying expression of gene markers of unknown clusters from May-Zhang et al., 2021 for both May-Zhang et al., 2021 datasets and the Morarach et al., 2021 juvenile dataset. D Expression of glial-like markers in the Wright et al., 2021 dataset on a dot plot. E Pre- and F post-integration PCA, showing proper integration of enteric neuron datasets. G Pre-integration UMAP of the enteric neuron meta-atlas.

Supplementary Material 2. Supplementary Figure 2. Muscle-like and enteric mesothelial fibroblast gene expression in the juvenile and adult meta-atlas. A Dot plot displaying expression of “muscle” expression markers in the combined juvenile and adult meta-atlas. B UMAPs showing expression of genes from A in the adult meta-atlas split by dataset, which identifies May-Zhang et al., 2021 as the main source of this gene expression. C-F UMAPs showing expression of genes from A in the May-Zhang et al., 2021 10X (C), InDrop (D), Morarach et al., 2021 (E), and Drokhlyansky et al., 2020 (F). G Dot plot showing expression of enteric mesothelial fibroblast marker genes identified in Zeisel et al., 2018 in clusters of the adult meta-atlas.

Supplementary Material 3. Supplementary Figure 3. Sex-biased gene expression of *Ddx3y*, *Uty*, *Tsix*, and *Xist* in each sc/snRNA-seq component datasets. A Heatmap of expression of sex-biased genes in MIRACL-seq colon neuronal cells from Drokhlyansky et al., 2020 split by mouse. B Heatmap of expression of sex-biased genes in MIRACL-seq Ileum neuronal cells from Drokhlyansky et al., 2020 split by mouse. C Heatmap of expression of sex-biased genes in Morarach et al., 2021 split by scRNA-seq run. D Heatmap of expression of sex-biased genes in RAISIN-seq colon neuronal cells from Drokhlyansky et al., 2020 split by mouse. E Heatmap of expression of sex-biased genes in May-Zhang et al., 2021 10X data split by snRNA-seq run. F Heatmap of expression of sex-biased genes in Wright et al., 2021 split by snRNA-seq run. G Heatmap of expression of sex-biased genes in May-Zhang et al., 2021 InDrop data split by snRNA-seq run. H Heatmap of expression of sex-biased genes in Zeisel et al., 2018 split by snRNA-seq run.

Supplementary Material 4. Supplementary Figure 4. Consistency of gene expression differentially expressed by sex across dataset and tissue segment. A Violin plots showing expression of known sex-specific genes *Ddx3y*, *Eif2s3y*, *Uty*, *Kdm5d*, *Tsix*, and *Xist* split by dataset of origin and tissue segment. B Violin plots showing expression of “*Gm*-” prefix genes split by dataset of origin and tissue segment. C Violin plots showing expression of annotated genes differentially expressed by sex split by dataset of origin and tissue segment. Red indicates female (left in each comparison) while blue indicates male (right in each comparison). May-Zhang InDrop Ileum only contains male cells and therefore comparisons cannot be made with female for this segment from this specific dataset.

Supplementary Material 5. Supplementary Table 1. May-Zhang2021_10x_6wks_MouseColDuodIle_SCTv2Integrated_FAM.

Supplementary Material 6. Supplementary Table 2. May-Zhang2021_InDrop_6wks_MouseColDuodIle_SCTv2Integrated_FAM.

Supplementary Material 7. Supplementary Table 3. Morarach2021_P21_MouseSmallIntestine_SCTv2Integrated_FAM.

Supplementary Material 8. Supplementary Table 4. DrokhlyanskyNeuronsCombined_SCTv2Integrated_FAM.

Supplementary Material 9. Supplementary Table 5. Wright2021_MADC_50DaysOld_AllData_SCTv2Integrated_FAM.

Supplementary Material 10. Supplementary Table 6. Wright2021_MADC_50DaysOld_NeuronClusters_SCTv2Integrated_FAM.

Supplementary Material 11. Supplementary Table 7. Zeisel2018_P19P21P23_NeuronsOnly_2_MouseSmallIntestine_SCTv2BatchCorrected_FAM.

Supplementary Material 12. Supplementary Table 8. ENIntegrated_FindAllMarkersPosValuesFCFilt_RNA_Assay.

Supplementary Material 13. Supplementary Table 9. ADULT_ENIntegrated_FindAllMarkersPosValuesFCFilt_RNA_Assay.

Supplementary Material 14. Supplementary Table 10. ADULT_Bnc2plusEtv1vstherest_fcfilt_FindMarkers_RNA_Assay.

Supplementary Material 15. Supplementary Table 11. ADULT_ENC12_FAM14vs21_FindMarkers_RNA_Assay.

Supplementary Material 16. Supplementary Table 12. ADULT_Nmu_FAM5vs16_FindMarkers_RNA_Assay.

Supplementary Material 17. Supplementary Table 13. Adult_NoWright_LROutDataset_BySexFindMarkers_RNA_Assay_SigFilt.

## Data Availability

The R code used to produce the Meta-Atlas in.R format, our reprocessing of each dataset used in the construction of the Meta-Atlas, the Meta-Atlas consisting of the adult + juvenile datasets, and the adult-only Meta-Atlas dataset are available as Seurat Objects for download on Zenodo at [https://doi.org/10.5281/zenodo.17420912] (https:/doi.org/10.5281/zenodo.17420912). Original datasets for the meta-atlas are accessible as follows Zeisel 2018 datasets (http://mousebrain.org/adolescent/tissues.html under “Enteric”); Drokhlyansky 2020 datasets (https://singlecell.broadinstitute.org/single_cell under accession number SCP1038); May-Zhang 2021 datasets (Gene Expression Omnibus under accession number GSE153202); Morarach 2021 datasets (Gene Expression Omnibus under accession number GSE149524); and Wright 2021 datasets (Gene Expression Omnibus under accession number GSE156905).

## Abbreviations

dpc: Days post coitus
ENS: Enteric Nervous System
ENC: Enteric Neuron Cluster
FACS: Fluorescence activated cell sorting
GEO: Gene Expression Omnibus
GI: Gastrointestinal
P: Postnatal
scRNA-seq: Single cell RNA-sequencing
snRNA-seq: Single nucleus RNA-sequencing
UMAP: Uniform manifold approximation projection
WT: wild type

## References

[1] Furness JB. The enteric nervous system and neurogastroenterology. Nat Rev Gastroenterol Hepatol. 2012;9(5):286–94. 22392290 10.1038/nrgastro.2012.32

[2] Dharshika C, Gulbransen BD. Enteric neuromics: how high-throughput “omics” deepens our understanding of enteric nervous system genetic architecture. Cell Mol Gastroenterol Hepatol. 2023;15(2):487–504. 36368612 10.1016/j.jcmgh.2022.10.019 PMC9792566

[3] Musser MA, Correa H, Southard-Smith EM. Enteric neuron imbalance and proximal dysmotility in ganglionated intestine of the Sox10(Dom/+) Hirschsprung mouse model. Cell Mol Gastroenterol Hepatol. 2015;1(1):87–101. 25844395 10.1016/j.jcmgh.2014.08.002 PMC4380251

[4] Cheng LS, Schwartz DM, Hotta R, Graham HK, Goldstein AM. Bowel dysfunction following pullthrough surgery is associated with an overabundance of nitrergic neurons in Hirschsprung disease. J Pediatr Surg. 2016;51(11):1834–8. 27570241 10.1016/j.jpedsurg.2016.08.001 PMC5065396

[5] Bhave S, Guyer RA, Picard N, Omer M, Hotta R, Goldstein AM. Ednrb (-/-) mice with hirschsprung disease are missing Gad2-expressing enteric neurons in the ganglionated small intestine. Front Cell Dev Biol. 2022;10:917243. 35959491 10.3389/fcell.2022.917243 PMC9360620

[6] Drokhlyansky E, Smillie CS, Van Wittenberghe N, Ericsson M, Griffin GK, Eraslan G, et al. The human and mouse enteric nervous system at single-cell resolution. Cell. 2020;182(6):1606-1622 e1623. 32888429 10.1016/j.cell.2020.08.003 PMC8358727

[7] Lai FP, Li Z, Zhou T, Leung AOW, Lau ST, Lui KN, et al. Ciliary protein Kif7 regulates Gli and Ezh2 for initiating the neuronal differentiation of enteric neural crest cells during development. Sci Adv. 2021;7(42):eabf7472. 34644112 10.1126/sciadv.abf7472 PMC8514102

[8] Lasrado R, Boesmans W, Kleinjung J, Pin C, Bell D, Bhaw L, et al. Lineage-dependent spatial and functional organization of the mammalian enteric nervous system. Science. 2017;356(6339):722–6. 28522527 10.1126/science.aam7511

[9] Lau ST, Li Z, Pui-Ling Lai F, Nga-Chu Lui K, Li P, Munera JO, et al. Activation of hedgehog signaling promotes development of mouse and human enteric neural crest cells based on single-cell transcriptome analyses. Gastroenterology. 2019;157(6):1556-1571 e1555. 31442438 10.1053/j.gastro.2019.08.019

[10] May-Zhang AA, Tycksen E, Southard-Smith AN, Deal KK, Benthal JT, Buehler DP, et al. Combinatorial transcriptional profiling of mouse and human enteric neurons identifies shared and disparate subtypes in situ. Gastroenterology. 2021;160(3):755-770 e726. 33010250 10.1053/j.gastro.2020.09.032 PMC7878294

[11] Morarach K, Mikhailova A, Knoflach V, Memic F, Kumar R, Li W, et al. Diversification of molecularly defined myenteric neuron classes revealed by single-cell RNA sequencing. Nat Neurosci. 2021;24(1):34–46. 33288908 10.1038/s41593-020-00736-x PMC7610403

[12] Wright CM, Schneider S, Smith-Edwards KM, Mafra F, Leembruggen AJL, Gonzalez MV, et al. scRNA-Seq Reveals New Enteric Nervous System Roles for GDNF, NRTN, and TBX3. Cell Mol Gastroenterol Hepatol. 2021;11(5):1548-1592 e1541. 33444816 10.1016/j.jcmgh.2020.12.014 PMC8099699

[13] Zeisel A, Hochgerner H, Lonnerberg P, Johnsson A, Memic F, van der Zwan J, et al. Molecular architecture of the mouse nervous system. Cell. 2018;174(4):999-1014 e1022. 30096314 10.1016/j.cell.2018.06.021 PMC6086934

[14] Guyer RA, Stavely R, Robertson K, Bhave S, Mueller JL, Picard NM, et al. Single-cell multiome sequencing clarifies enteric glial diversity and identifies an intraganglionic population poised for neurogenesis. Cell Rep. 2023;42(3):112194. 36857184 10.1016/j.celrep.2023.112194 PMC10123761

[15] Vincent E, Chatterjee S, Cannon GH, Auer D, Ross H, Chakravarti A, et al. Ret deficiency decreases neural crest progenitor proliferation and restricts fate potential during enteric nervous system development. Proc Natl Acad Sci U S A. 2023;120(34):e2211986120. 37585461 10.1073/pnas.2211986120 PMC10451519

[16] Kulkarni S, Saha M, Slosberg J, Singh A, Nagaraj S, Becker L, Zhang C, Bukowski A, Wang Z, Liu G et al: Age-associated changes in lineage composition of the enteric nervous system regulate gut health and disease. Elife 2023;12. 10.7554/eLife.88051 PMC10727506 38108810

[17] Schneider S, Anderson JB, Bradley RP, Beigel K, Wright CM, Maguire BA, Yan G, Taylor DM, Harbour JW, Heuckeroth RO: BAP1 is required prenatally for differentiation and maintenance of postnatal murine enteric nervous system. J Clin Invest 2024, 134(9). 10.1172/JCI177771 PMC11060734 38690732

[18] Zhou B, Feng C, Sun S, Chen X, Zhuansun D, Wang D, et al. Identification of signaling pathways that specify a subset of migrating enteric neural crest cells at the wavefront in mouse embryos. Dev Cell. 2024;59(13):1689-1706 e1688. 38636517 10.1016/j.devcel.2024.03.034

[19] Li W, Morarach K, Liu Z, Banerjee S, Chen Y, Harb AL, et al. The transcriptomes, connections and development of submucosal neuron classes in the mouse small intestine. Nat Neurosci. 2025;28(6):1146–59. 40442499 10.1038/s41593-025-01962-x PMC12148937

[20] Li Z, Ngan ES. New insights empowered by single-cell sequencing: from neural crest to enteric nervous system. Comput Struct Biotechnol J. 2022;20:2464–72. 35664232 10.1016/j.csbj.2022.05.025 PMC9133688

[21] Guyer RA, Mueller JL, Goldstein AM: Applications of single-cell sequencing technology to the enteric nervous system. Biomolecules 2022;12(3). 10.3390/biom12030452 PMC8946246 35327644

[22] Young HM, Bergner AJ, Muller T. Acquisition of neuronal and glial markers by neural crest-derived cells in the mouse intestine. J Comp Neurol. 2003;456(1):1–11. 12508309 10.1002/cne.10448

[23] Karkala F, de Bosscher I, Windster JD, Stroebel S, van Zanten L, Alves MM, Sacchetti A: Flow cytometric analysis and sorting of murine enteric nervous system cells: an optimized protocol. Int J Mol Sci 2025;26(10). 10.3390/ijms26104824 PMC12112686 40429965

[24] Windster JD, Sacchetti A, Schaaf GJ, Bindels EM, Hofstra RM, Wijnen RM, et al. A combinatorial panel for flow cytometry-based isolation of enteric nervous system cells from human intestine. EMBO Rep. 2023;24(4):e55789. 36852936 10.15252/embr.202255789 PMC10074091

[25] Corpening JC, Cantrell VA, Deal KK, Southard-Smith EM. A Histone2BCerulean BAC transgene identifies differential expression of Phox2b in migrating enteric neural crest derivatives and enteric glia. Dev Dyn. 2008;237(4):1119–32. 18351668 10.1002/dvdy.21498 PMC3093109

[26] Choi HMT, Schwarzkopf M, Fornace ME, Acharya A, Artavanis G, Stegmaier J, Cunha A, Pierce NA: Third-generation in situ hybridization chain reaction: multiplexed, quantitative, sensitive, versatile, robust. Development 2018;145(12). 10.1242/dev.165753 PMC6031405 29945988

[27] Choi HMT, Schwarzkopf M, Pierce NA. Multiplexed quantitative in situ hybridization with subcellular or single-molecule resolution within whole-mount vertebrate embryos: qHCR and dHCR Imaging (v30). Methods Mol Biol. 2020;2148:159–78. 32394381 10.1007/978-1-0716-0623-0_10

[28] May-Zhang AA, Benthal JT, Southard-Smith EM. Hybridization chain reaction for mRNA localization in single cells from mouse and human cryosections. Curr Protoc. 2022;2(5):e439. 35612422 10.1002/cpz1.439 PMC9202517

[29] Garg M, Li X, Moreno P, Papatheodorou I, Shu Y, Brazma A, et al. Meta-analysis of COVID-19 single-cell studies confirms eight key immune responses. Sci Rep. 2021;11(1):20833. 34675242 10.1038/s41598-021-00121-z PMC8531356

[30] Rocque B, Barbetta A, Singh P, Goldbeck C, Helou DG, Loh YE, et al. Creation of a single cell RNAseq meta-atlas to define human liver immune homeostasis. Front Immunol. 2021;12:679521. 34335581 10.3389/fimmu.2021.679521 PMC8322955

[31] Zernecke A, Winkels H, Cochain C, Williams JW, Wolf D, Soehnlein O, et al. Meta-analysis of leukocyte diversity in atherosclerotic mouse aortas. Circ Res. 2020;127(3):402–26. 32673538 10.1161/CIRCRESAHA.120.316903 PMC7371244

[32] Zheng GX, Terry JM, Belgrader P, Ryvkin P, Bent ZW, Wilson R, et al. Massively parallel digital transcriptional profiling of single cells. Nat Commun. 2017;8:14049. 28091601 10.1038/ncomms14049 PMC5241818

[33] Satija R, Farrell JA, Gennert D, Schier AF, Regev A. Spatial reconstruction of single-cell gene expression data. Nat Biotechnol. 2015;33(5):495–502. 25867923 10.1038/nbt.3192 PMC4430369

[34] Butler A, Hoffman P, Smibert P, Papalexi E, Satija R. Integrating single-cell transcriptomic data across different conditions, technologies, and species. Nat Biotechnol. 2018;36(5):411–20. 29608179 10.1038/nbt.4096 PMC6700744

[35] Stuart T, Butler A, Hoffman P, Hafemeister C, Papalexi E, Mauck WM 3rd, et al. Comprehensive integration of single-cell data. Cell. 2019;177(7):1888-1902 e1821. 31178118 10.1016/j.cell.2019.05.031 PMC6687398

[36] Hao Y, Hao S, Andersen-Nissen E, Mauck WM 3rd, Zheng S, Butler A, et al. Integrated analysis of multimodal single-cell data. Cell. 2021;184(13):3573-3587 e3529. 34062119 10.1016/j.cell.2021.04.048 PMC8238499

[37] Hao Y, Stuart T, Kowalski MH, Choudhary S, Hoffman P, Hartman A, et al. Dictionary learning for integrative, multimodal and scalable single-cell analysis. Nat Biotechnol. 2024;42(2):293–304. 37231261 10.1038/s41587-023-01767-y PMC10928517

[38] The Mouse Brain Atlas [https://storage.googleapis.com/linnarsson-lab-loom/l1_enteric.loom]. Accessed 02 July 2024.

[39] Hafemeister C, Satija R. Normalization and variance stabilization of single-cell RNA-seq data using regularized negative binomial regression. Genome Biol. 2019;20(1):296. 31870423 10.1186/s13059-019-1874-1 PMC6927181

[40] Choudhary S, Satija R. Comparison and evaluation of statistical error models for scRNA-seq. Genome Biol. 2022;23(1):27. 35042561 10.1186/s13059-021-02584-9 PMC8764781

[41] Chazarra-Gil R, van Dongen S, Kiselev VY, Hemberg M. Flexible comparison of batch correction methods for single-cell RNA-seq using BatchBench. Nucleic Acids Res. 2021;49(7):e42. 33524142 10.1093/nar/gkab004 PMC8053088

[42] Miller SA, Policastro RA, Sriramkumar S, Lai T, Huntington TD, Ladaika CA, et al. LSD1 and aberrant DNA methylation mediate persistence of enteroendocrine progenitors that support BRAF-mutant colorectal cancer. Cancer Res. 2021;81(14):3791–805. 34035083 10.1158/0008-5472.CAN-20-3562 PMC8513805

[43] Borghini S, Di Duca M, Santamaria G, Vargiolu M, Bachetti T, Cargnin F, et al. Transcriptional regulation of TLX2 and impaired intestinal innervation: possible role of the PHOX2A and PHOX2B genes. Eur J Hum Genet. 2007;15(8):848–55. 17505528 10.1038/sj.ejhg.5201852

[44] Borghini S, Bachetti T, Fava M, Di Duca M, Cargnin F, Fornasari D, et al. The TLX2 homeobox gene is a transcriptional target of PHOX2B in neural-crest-derived cells. Biochem J. 2006;395(2):355–61. 16402914 10.1042/BJ20051386 PMC1422762

[45] Parisi MA, Baldessari AE, Iida MH, Clarke CM, Doggett B, Shirasawa S, et al. Genetic background modifies intestinal pseudo-obstruction and the expression of a reporter gene in *Hox11L1*-/- mice. Gastroenterology. 2003;125(5):1428–40. 14598259 10.1016/j.gastro.2003.08.021

[46] Hatano M, Aoki T, Dezawa M, Yusa S, Iitsuka Y, Koseki H, et al. A novel pathogenesis of megacolon in Ncx/Hox11L.1 deficient mice. J Clin Invest. 1997;100(4):795–801. 9259577 10.1172/JCI119593 PMC508250

[47] Shirasawa S, Yunker AM, Roth KA, Brown GA, Horning S, Korsmeyer SJ. Enx (Hox11L1)-deficient mice develop myenteric neuronal hyperplasia and megacolon. Nat Med. 1997;3(6):646–50. 9176491 10.1038/nm0697-646

[48] Ghaleb AM, Bialkowska AB, Snider AJ, Gnatenko DV, Hannun YA, Yang VW, et al. IQ motif-containing GTPase-activating protein 2 (IQGAP2) is a novel regulator of colonic inflammation in mice. PLoS ONE. 2015;10(6):e0129314. 26047140 10.1371/journal.pone.0129314 PMC4457730

[49] Bae H, Kim B, Lee H, Lee S, Kang HS, Kim SJ. Epigenetically regulated fibronectin leucine rich transmembrane protein 2 (FLRT2) shows tumor suppressor activity in breast cancer cells. Sci Rep. 2017;7(1):272. 28325946 10.1038/s41598-017-00424-0 PMC5428463

[50] Li J, Shinoda Y, Ogawa S, Ikegaya S, Li S, Matsuyama Y, et al. Expression of FLRT2 in postnatal central nervous system development and after spinal cord injury. Front Mol Neurosci. 2021;14:756264. 34744626 10.3389/fnmol.2021.756264 PMC8569257

[51] Katzeff JS, Lok HC, Bhatia S, Fu Y, Halliday GM, Kim WS. ATP-binding cassette transporter expression is widely dysregulated in frontotemporal dementia with TDP-43 inclusions. Front Mol Neurosci. 2022;15:1043127. 36385764 10.3389/fnmol.2022.1043127 PMC9663841

[52] Avetisyan M, Wang H, Schill EM, Bery S, Grider JR, Hassell JA, et al. Hepatocyte growth factor and MET support mouse enteric nervous system development, the peristaltic response, and intestinal epithelial proliferation in response to injury. J Neurosci. 2015;35(33):11543–58. 26290232 10.1523/JNEUROSCI.5267-14.2015 PMC4540795

[53] Mouse brain atlas cell types: ENT9 [http://mousebrain.org/celltypes/ENT9.html]. Accessed 02 July 2024.

[54] Klein AM, Mazutis L, Akartuna I, Tallapragada N, Veres A, Li V, et al. Droplet barcoding for single-cell transcriptomics applied to embryonic stem cells. Cell. 2015;161(5):1187–201. 26000487 10.1016/j.cell.2015.04.044 PMC4441768

[55] Shin D, Lee W, Lee JH, Bang D. Multiplexed single-cell RNA-seq via transient barcoding for simultaneous expression profiling of various drug perturbations. Sci Adv. 2019;5(5):eaav2249. 31106268 10.1126/sciadv.aav2249 PMC6520024

[56] Rademakers G, Vaes N, Schonkeren S, Koch A, Sharkey KA, Melotte V. The role of enteric neurons in the development and progression of colorectal cancer. Biochim Biophys Acta Rev Cancer. 2017;1868(2):420–34. 28847715 10.1016/j.bbcan.2017.08.003

[57] Ray K. Crosstalk between enteric neurons and colorectal cancer stem cells influences self-renewal. Nat Rev Gastroenterol Hepatol. 2022;19(7):416. 35668161 10.1038/s41575-022-00641-7

[58] Clark IC, Fontanez KM, Meltzer RH, Xue Y, Hayford C, May-Zhang A, et al. Microfluidics-free single-cell genomics with templated emulsification. Nat Biotechnol. 2023;41(11):1557–66. 36879006 10.1038/s41587-023-01685-z PMC10635830

[59] Fuzik J, Zeisel A, Mate Z, Calvigioni D, Yanagawa Y, Szabo G, et al. Integration of electrophysiological recordings with single-cell RNA-seq data identifies neuronal subtypes. Nat Biotechnol. 2016;34(2):175–83. 26689544 10.1038/nbt.3443 PMC4745137

[60] Chen W, Guillaume-Gentil O, Rainer PY, Gabelein CG, Saelens W, Gardeux V, et al. Live-seq enables temporal transcriptomic recording of single cells. Nature. 2022;608(7924):733–40. 35978187 10.1038/s41586-022-05046-9 PMC9402441

[61] Sikkema L, Ramirez-Suastegui C, Strobl DC, Gillett TE, Zappia L, Madissoon E, et al. An integrated cell atlas of the lung in health and disease. Nat Med. 2023;29(6):1563–77. 37291214 10.1038/s41591-023-02327-2 PMC10287567

[62] Picelli S, Bjorklund AK, Faridani OR, Sagasser S, Winberg G, Sandberg R. Smart-seq2 for sensitive full-length transcriptome profiling in single cells. Nat Methods. 2013;10(11):1096–8. 24056875 10.1038/nmeth.2639

[63] McGinnis CS, Murrow LM, Gartner ZJ. DoubletFinder: doublet detection in single-cell RNA sequencing data using artificial nearest neighbors. Cell Syst. 2019;8(4):329-337 e324. 30954475 10.1016/j.cels.2019.03.003 PMC6853612

[64] 10X Genomics, What is the maximum number of cells that can be profiled? [https://kb.10xgenomics.com/hc/en-us/articles/360001378811-What-is-the-maximum-number-of-cells-that-can-be-profiled]. Accessed 02 July 2024.

