# Supplementary figures and images for: Building consensus: construction of a juvenile and adult scRNA-seq meta-atlas for dataset comparisons and harmonizing transcriptomic definitions of enteric neuron subtypes
Source: BMC Genomics. 2026 Jan 22;27:50. doi: 10.1186/s12864-025-12283-5 (PMC12825290; doi:10.1186/s12864-025-12283-5)

SupFigure1

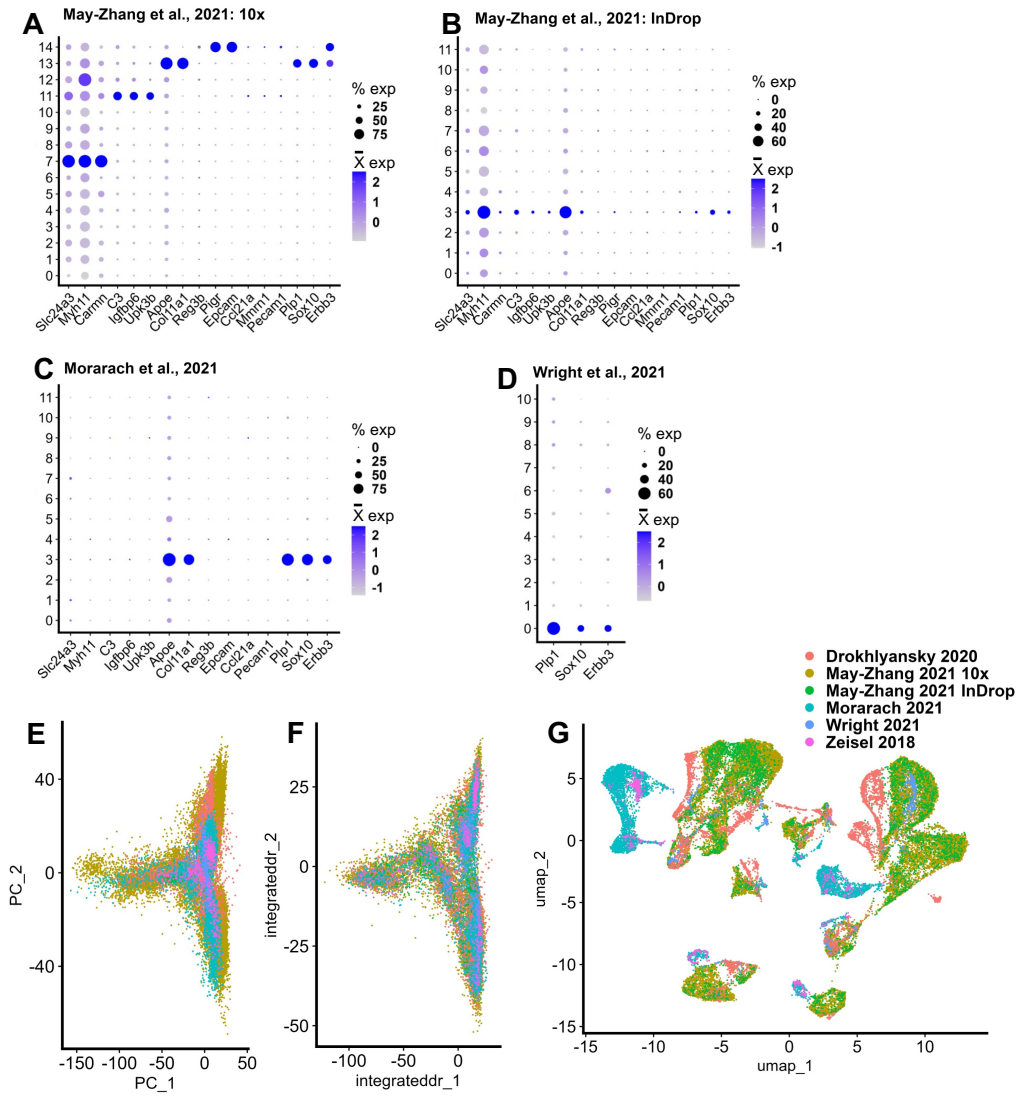

Supplement: Supplementary file 1 — Supplementary Material 1. Supplementary Figure 1. Identification of putative non-neuronal clusters to remove for effective meta-atlas generation and batch correction. A-C Dot plots displaying expression of gene markers of unknown clusters from May-Zhang et al., 2021 for both May-Zhang et al., 2021 datasets and the Morarach et al., 2021 juvenile dataset. D Expression of glial-like markers in the Wright et al., 2021 dataset on a dot plot. E Pre- and F post-integration PCA, showing proper integration of enteric neuron datasets. G Pre-integration UMAP of the enteric neuron meta-atlas. [file 12864_2025_12283_MOESM1_ESM.pdf]

# SupFigure2

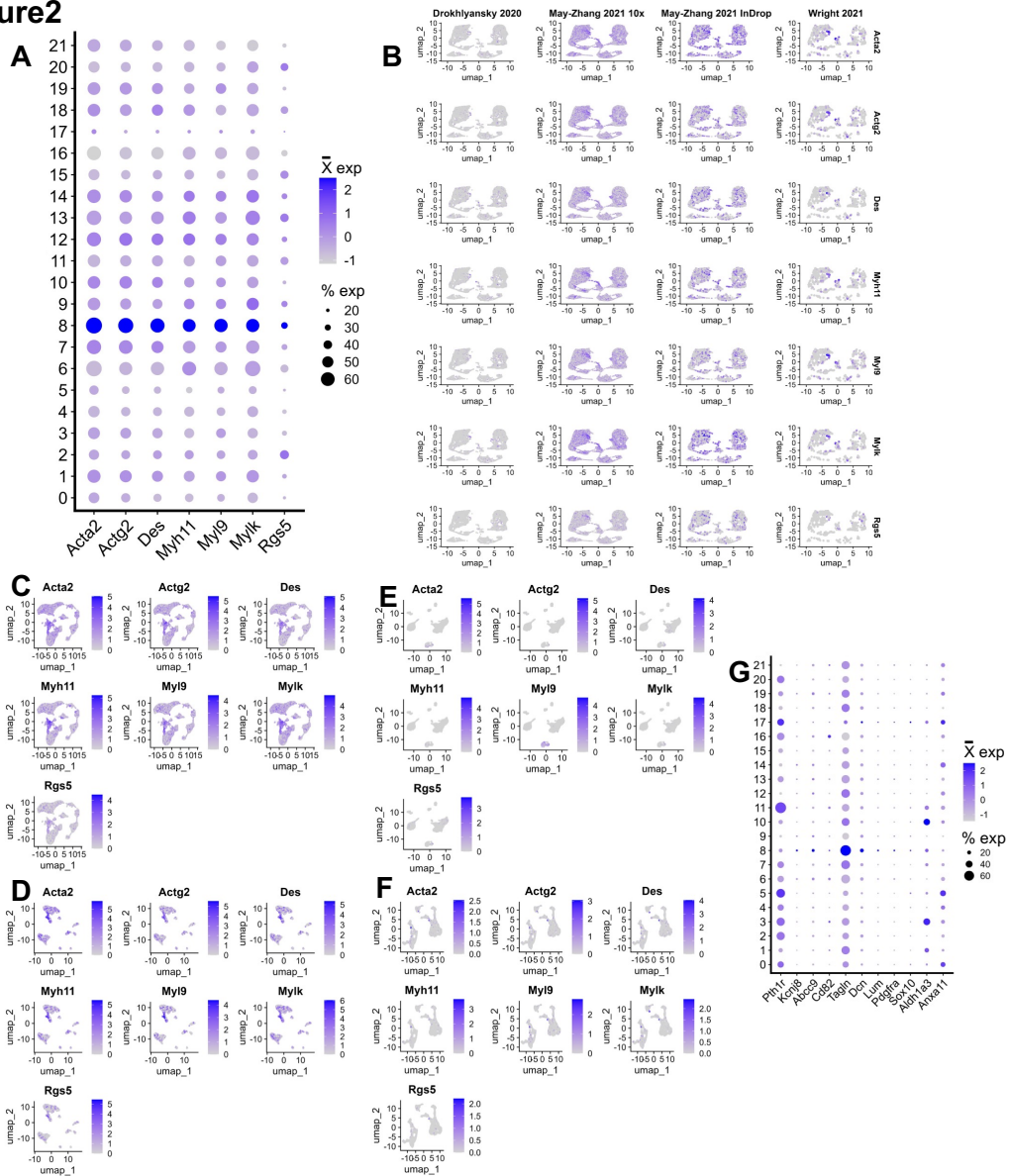

Supplement: Supplementary file 2 — Supplementary Material 2. Supplementary Figure 2. Muscle-like and enteric mesothelial fibroblast gene expression in the juvenile and adult meta-atlas. A Dot plot displaying expression of “muscle” expression markers in the combined juvenile and adult meta-atlas. B UMAPs showing expression of genes from A in the adult meta-atlas split by dataset, which identifies May-Zhang et al., 2021 as the main source of this gene expression. C-F UMAPs showing expression of genes from A in the May-Zhang et al., 2021 10X (C), InDrop (D), Morarach et al., 2021 (E), and Drokhlyansky et al., 2020 (F). G Dot plot showing expression of enteric mesothelial fibroblast marker genes identified in Zeisel et al., 2018 in clusters of the adult meta-atlas. [file 12864_2025_12283_MOESM2_ESM.pdf]

SupFigure3

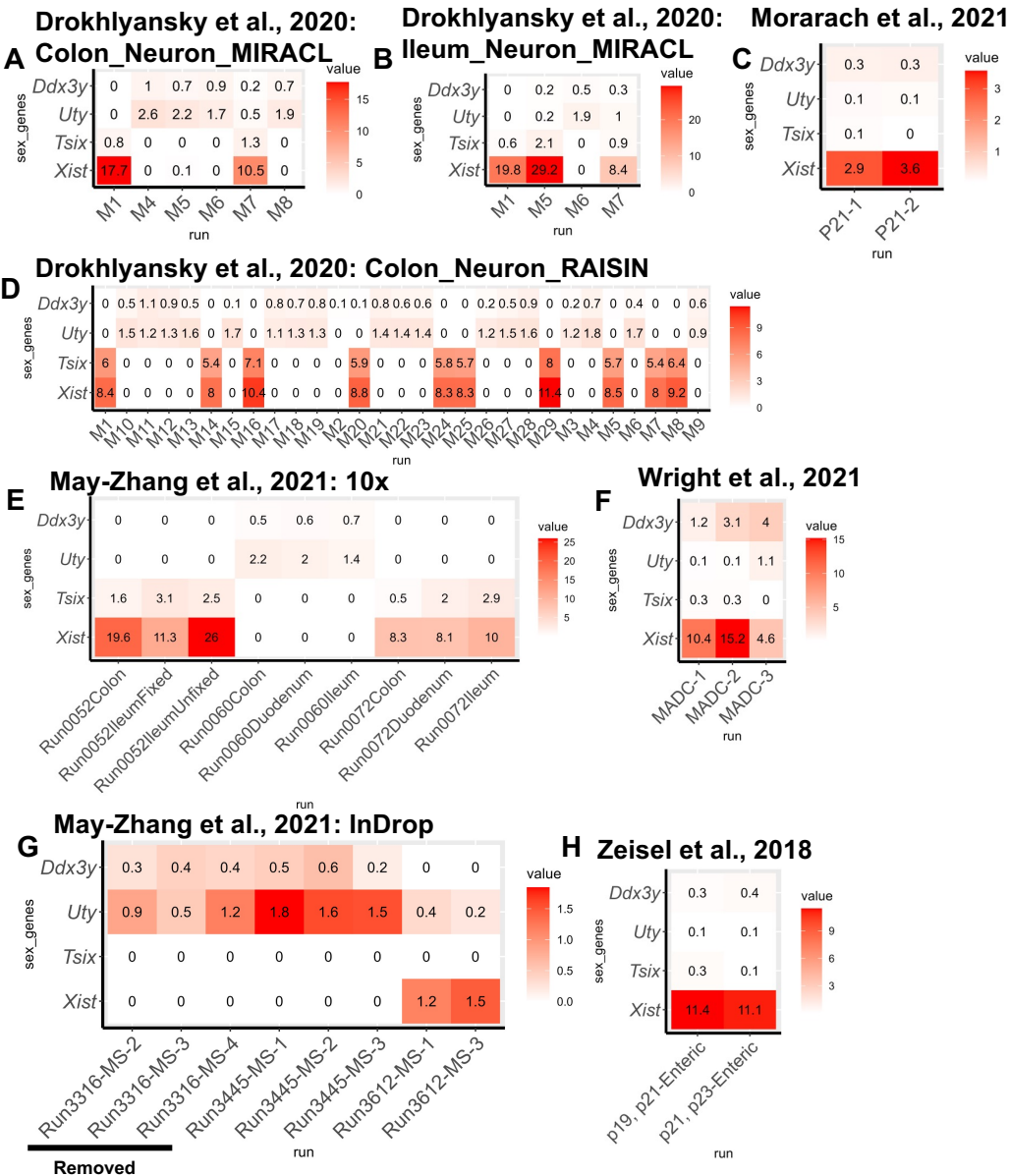

Supplement: Supplementary file 3 — Supplementary Material 3. Supplementary Figure 3. Sex-biased gene expression of Ddx3y, Uty, Tsix, and Xist in each sc/snRNA-seq component datasets. A Heatmap of expression of sex-biased genes in MIRACL-seq colon neuronal cells from Drokhlyansky et al., 2020 split by mouse. B Heatmap of expression of sex-biased genes in MIRACL-seq Ileum neuronal cells from Drokhlyansky et al., 2020 split by mouse. C Heatmap of expression of sex-biased genes in Morarach et al., 2021 split by scRNA-seq run. D Heatmap of expression of sex-biased genes in RAISIN-seq colon neuronal cells from Drokhlyansky et al., 2020 split by mouse. E Heatmap of expression of sex-biased genes in May-Zhang et al., 2021 10X data split by snRNA-seq run. F Heatmap of expression of sex-biased genes in Wright et al., 2021 split by snRNA-seq run. G Heatmap of expression of sex-biased genes in May-Zhang et al., 2021 InDrop data split by snRNA-seq run. H Heatmap of expression of sex-biased genes in Zeisel et al., 2018 split by snRNA-seq run. [file 12864_2025_12283_MOESM3_ESM.pdf]

SupFigure4

A

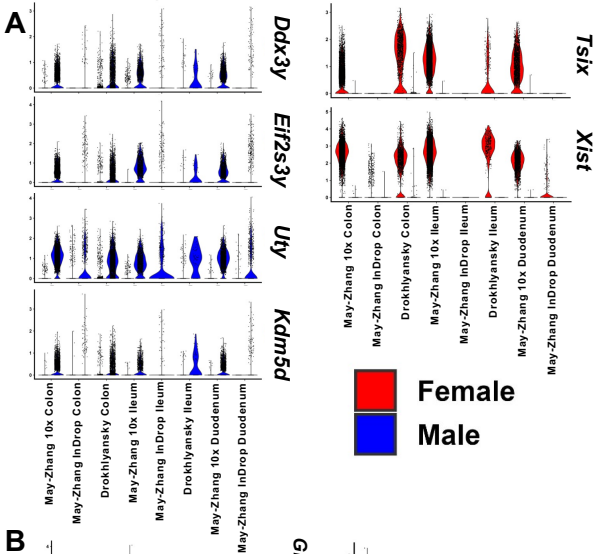

B

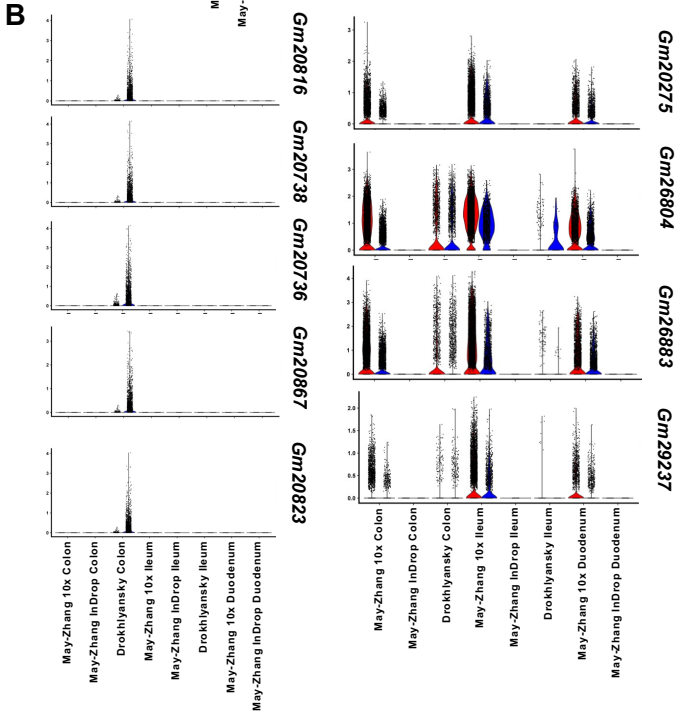

C

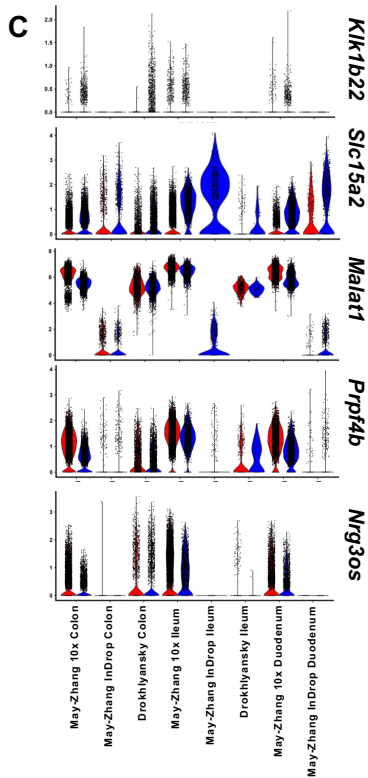

Supplement: Supplementary file 4 — Supplementary Material 4. Supplementary Figure 4. Consistency of gene expression differentially expressed by sex across dataset and tissue segment. A Violin plots showing expression of known sex-specific genes Ddx3y, Eif2s3y, Uty, Kdm5d, Tsix, and Xist split by dataset of origin and tissue segment. B Violin plots showing expression of “Gm-” prefix genes split by dataset of origin and tissue segment. C Violin plots showing expression of annotated genes differentially expressed by sex split by dataset of origin and tissue segment. Red indicates female (left in each comparison) while blue indicates male (right in each comparison). May-Zhang InDrop Ileum only contains male cells and therefore comparisons cannot be made with female for this segment from this specific dataset. [file 12864_2025_12283_MOESM4_ESM.pdf]
